# Mitotic exchange in female germline stem cells is the major source of *Sex Ratio* chromosome recombination in *Drosophila pseudoobscura*

**DOI:** 10.1093/g3journal/jkac264

**Published:** 2022-10-04

**Authors:** Spencer Koury

**Affiliations:** Stowers Institute for Medical Research, Kansas City, MO 64110, USA; School of Biological Sciences, University of Utah, Salt Lake City, UT 84112, USA

**Keywords:** *Drosophila pseudoobscura*, mitotic exchange, *Sex Ratio*, germline stem cells

## Abstract

*Sex Ratio* chromosomes in *Drosophila pseudoobscura* are selfish *X* chromosome variants associated with 3 nonoverlapping inversions. In the male germline, *Sex Ratio* chromosomes distort the segregation of *X* and *Y* chromosomes (99:1), thereby skewing progeny sex ratio. In the female germline, segregation of *Sex Ratio* chromosomes is mendelian (50:50), but nonoverlapping inversions strongly suppress recombination establishing a 26-Mb haplotype (constituting ∼20% of the haploid genome). Rare crossover events located between nonoverlapping inversions can disrupt this haplotype, and recombinants have sometimes been found in natural populations. We recently reported on the first lab-generated *Sex Ratio* recombinants occurring at a rate of 0.0012 crossovers per female meiosis. An improved experimental design presented here reveals that these recombination events were at least 4 times more frequent than previously estimated. Furthermore, recombination events were strongly clustered, indicating that the majority arose from mitotic exchange in female germline stem cells and not from meiotic crossing-over in primary oocytes. Finally, asymmetric recovery of complementary recombinants was consistent with unequal exchange causing the recombination-induced viability defects. Incorporating these experimental results into population models for *Sex Ratio* chromosome evolution provided a substantially better fit to natural population frequencies and allowed maintenance of the highly differentiated 26-Mb *Sex Ratio* haplotype without invoking strong epistatic selection. This study provides the first estimate of spontaneous mitotic exchange for naturally occurring chromosomes in *Drosophila* female germline stem cells, reveals a much higher *Sex Ratio* chromosome recombination rate, and develops a mathematical model that accurately predicts the rarity of recombinant *Sex Ratio* chromosomes in natural populations.

## Introduction

**Fig. 1.**
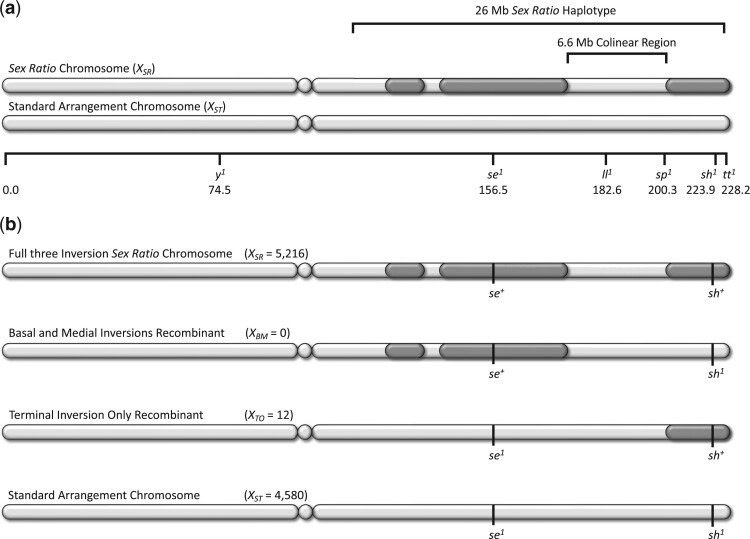
Schematic representation for recombination experiments with metacentric *X* chromosomes of *D. pseudoobscura*. a) Centromeres are depicted as centrally placed circles and the 3 nonoverlapping inverted regions of the *Sex Ratio* chromosomes are shown in dark gray on the *X* chromosome right arm. Physical dimensions of *Sex Ratio* haplotype and colinear region are listed above the chromosome, below chromosomes are the genetic map position for the visible markers. b) Summary of Fuller *et al.* (2020) recombination experiment with raw counts provided in parentheses, the asymmetry of this result was very unexpected under a reciprocal-exchange model of meiotic crossing-over $\left(\chi_{\left[1\right]}^{\;2}=12,\;P=2.85\times 10^{-4}\right)$.

Selfish sex chromosomes are a special class of segregation distorters found in organisms with chromosomal sex determination (Burt and Trivers 2006). Because nonmendelian transmission of the sex chromosomes skews the ratio of female to male offspring, these chromosomal variants are called *Sex Ratio* chromosomes. *Sex Ratio* (*X_SR_*) chromosomes are found in 16 species of *Drosophila* (an *XY* sex-determination system) where they are typically associated with multiple inversions of the *X* chromosome (Jaenike 2001; Courret *et al.* 2019). In *Drosophila pseudoobscura*, all necessary and sufficient genes for the strong sex ratio distortion phenotype are linked to 3 nonoverlapping inversions of the right arm of the metacentric *X* chromosome (Fig. 1a) (Dobzhansky and Epling 1944; Wallace 1948).

In natural populations of *D. pseudoobscura*, the 3 inversions of *X_SR_* chromosomes are found in near perfect linkage disequilibrium $\left(r^{2}=0.99\right)$ generating a single, large, highly differentiated 26-Mb *Sex Ratio* (*SR*) haplotype (Lewontin 1964; Fuller *et al.* 2020). Rare recombinant chromosomes that disrupt the 3-inversion 26-Mb *SR* haplotype have been observed in natural population surveys, but only 5 instances have been found in cumulative sampling of greater than 10,000 *X* chromosomes (Wallace 1948; Beckenbach 1996; Fuller *et al.* 2020). Cytogenetic analysis confirms that these rare recombinants did not arise from ectopic exchange between *D. pseudoobscura X_SR_* and *Y* chromosomes; and more generally, brachycerous Dipterans have achiasmate male meiosis (Gethmann 1988; Beckenbach 1996). However, further detailed phenotypic study of recombinant *X_SR_* chromosomes has historically been complicated by their rarity and difficult maintenance in the lab (Wallace 1948).

We recently reported rare recombination (on the order of 1 in 1,000 female meioses) occurring in the 6.6-Mb colinear region located between the medial and terminal inversions for heterozygotes of *Sex Ratio* and *Standard X* chromosomes (*X_SR_/X_ST_*) (Fig. 1b) (Fuller *et al.* 2020). Segregation assays of recombinant chromosomes revealed that the *X*-linked genetic architecture of the strong sex ratio distortion phenotype consists of at least 2 loci: a distorter gene in the proximal half of the right arm and a modifier gene in the distal half of the right arm. Using a simple model of gametic phase disequilibrium, we inferred that strong epistatic selection must counteract recombination to maintain approximately 20% of the haploid genome as the single, highly differentiated *SR* haplotype observed in nature (Fuller *et al.* 2020). However, this conclusion was based on a model that did not account for the *X*-linked inheritance of *SR* haplotype, female-limited recombination or male-specific segregation distortion, and was highly sensitive to the experimentally determined recombination rate.

The recombination experiment reported in Fuller *et al.* (2020) followed standard testcross protocol by mass crossing 20 female *X_SR_/X_ST_* heterozygotes to 20 tester strain males in 6 fl. oz milk bottles (Bridges and Brehme 1944). For logistical reasons, only male progeny were scored for the recombination of *X*-linked visible markers before being discarded. Twelve of the nearly 10,000 males scored were recombinants; however, all 12 males exhibited the same marker combination (indicating a recombinant carrying only the terminal inversion of *X_SR_* chromosomes, denoted *X_TO_*) and we observed none of the complementary recombinant *X_SR_* chromosome (carrying both the basal and medial inversions but without the terminal inversion, denoted *X_BM_*). Under a reciprocal-exchange model of meiotic crossing-over, this was a very rare and unexpected result $\left(\chi_{\left[1\right]}^{\;2}=12,\;P=2.85\times 10^{-4}\right)$. Further screening (>10,000 individuals) demonstrated that stable *X_BM_* stocks could indeed be established, and polytene chromosome squashes confirmed that the recombination events occurred in the 6.6-Mb colinear region located between the medial and terminal inversions of the *SR* haplotype [see photomicrograph in Supplementary Fig. 7 of Fuller *et al.* (2020)].

In the Fuller *et al.* (2020) experimental design, the *short^1^* mutation marked the absence of the terminal inversion of *X_SR_* chromosomes. Only male progeny were scored for recombination due to incomplete penetrance of the wing vein mutation *short^1^* in female homozygotes. Any residual penetrance problems in hemizygous males could have caused incorrect classification of the missing recombinant *X_BM_* chromosomes as nonrecombinant wildtype *X_ST_* chromosomes. Furthermore, if the missing recombinant *X_BM_* chromosomes have strong recessive viability defects, then these recombinants would not have been detected in hemizygous male progeny. Finally, in a 10,000 fly experiment, other rare events (e.g. gene conversion or somatic mutation) could have inflated the estimated crossover rate. To address these potential sources of bias, a new recombination experiment was conducted using single-female crosses, scoring both male and female progeny with improved marker penetrance, and retaining recombinant progeny for confirmation testcrosses.

**Fig. 2.**
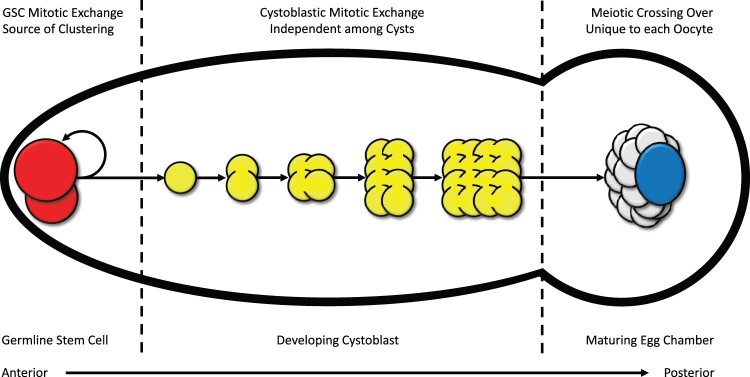
Cartoon sequence of germarium and single egg chamber development in anterior region of *Drosophila* ovariole. Mitotic exchange in GSCs (red on the far left) with their asymmetric division and self-renewal (reflexive arrow) can cause clustering in recombination datasets. Mitotic exchange in developing cystoblast (yellow in the center) cannot cause clustering as only 1 of 16 nuclei will develop into the oocyte (blue on the far right), with the other 15 becoming nurse cells (gray on the far right). Meiotic crossing-over in oocyte (blue) only occurs after development of the 16-cell cyst (yellow) and are causally independent among oocytes. Therefore, clustering of recombination events must originate from mitotic exchange in GSC or earlier in development. Cartoon does not represent a proportional scale but is meant to illustrate the sequential events allowing for interpretation of clustering in recombination data.

Beyond the potential design artifacts listed above, the rare *X_SR_*/*X_ST_* recombinants may not have been the product of meiotic crossing-over but rather arose from mitotic exchange in the female germline stem cells (GSCs) prior to meiosis. This biological source of variation would confound normal meiotic crossover rate estimation with a recombination process that precedes meiosis. In *Drosophila*, ovaries are divided into 16–20 ovarioles, each containing 2–3 GSCs occupying a niche in the anterior region of the ovariole (i.e. the germarium, as shown in Fig. 2). Self-renewing GSCs divide asymmetrically to produce a differentiating cystoblast, which undergoes 4 rounds of mitosis with incomplete cytokinesis to form a 16-cell cyst [reviewed in McLaughlin and Bratu (2015)]. In a well-studied determination process, only one of these 16 cells will develop into an oocyte and undergo meiosis while the other 15 cells become nurse cells that do not transmit genetic material to the next generation [reviewed in Huynh and St Johnston (2004)]. Therefore, recombinant progeny resulting from canonical meiotic crossing-over (reciprocal exchange) in each oocyte are causally independent of meiotic crossing-over in other oocytes. In contrast, mitotic exchange occurring in self-renewing stem cells has the potential to create clusters of nonindependent (clonal) recombinant progeny, all originating from a single GSC mitotic event (Fig. 2).

Mitotic exchange in the female germline cells is difficult to measure because crossing-over in prophase of meiosis I masks this signal under normal conditions (Ashburner *et al.* 2004). Nevertheless, 18% of *X-Y* exchanges from compound chromosome *C(1)RM/Y* in the female germline were attributed to GSC mitotic exchange (Neuhaus 1936), and rates of female GSC mitotic exchange have been elevated by X-ray irradiation (Bateman and Chandley 1965). In *D. pseudoobscura X_SR_*/*X_ST_* heterozygotes, the nonoverlapping inversions of *X_SR_* chromosomes strongly suppress meiotic crossing-over on the entire right arm of the *X* chromosome; thereby presenting a unique opportunity to measure spontaneous mitotic exchange in female GSCs for naturally occurring *X* chromosomes under normal conditions. In this specific context, GSC mitotic exchange can be detected statistically by the clustered distribution (i.e. overdispersion) of recombination events.

In this study, an improved experimental design revealed that recombination events were substantially more frequent than previously estimated. Statistical analysis rejected the hypothesis that meiotic crossing-over generated the present recombination dataset. Three distinct types of clustering in recombination events indicated that the nonindependence in this dataset arose from unequal mitotic exchange in the female GSCs. To understand the implications of these experimental results for natural populations, the Edwards (1961) population genetic model of *Sex Ratio* was extended to incorporate recombination in Appendix A. Modeling unequal mitotic exchange in the *Sex Ratio* system provided a substantially better fit to natural population frequencies of *X_SR_* chromosomes in *D. pseudoobscura* and, despite the higher recombination rate, the model was able to explain the relative rarity of recombinant *X_BM_* and *X_TO_* chromosomes in the wild.

## Materials and methods

### Live-stock construction

The 3 focal *Sex Ratio* (*X_SR_*) chromosomes were identified from natural population collections in Zion National Park, UT, USA, in September 2013 and Kaibab National Forest, AZ, USA, in September 2017. Subsequently, in 2018, the base *X_SR_* stocks were created by the reisolation of *X_SR_* chromosomes and 20 generations of backcrossing to 3 different highly inbred stocks (*F > *0.99), each with a multiply marked standard arrangement *X* chromosome (*X_ST_*). The resulting 9 stocks (3 *X_SR_* chromosome isolates on each of 3 different backgrounds) were segregating for an unmarked *X_SR_* and a marked *X_ST_* on a homozygous genetic background.

The visible markers of the *X_ST_* chromosomes spanned the whole right arm of the metacentric *X* chromosome; and in combination, the mutations *yellow^1^* (*y^1^*, 1–74.5), *sepia^1^* (*se^1^*, 1–156.5), *lanceolate^1^* (*ll^1^*, 1–182.6), *snapt^1^* (*sp^1^*, 1–200.3), *short^1^* (*sh^1^*, 1–225.9), and *tilt^1^* (*tt^1^*, 1–228.2) allowed detection of rare *X_SR_* recombinant events (Orr 1995; Fuller *et al.* 2020). The marker penetrance of *sh^1^* in this study was significantly improved through 2 additional years of inbreeding and marker selection subsequent to the Fuller *et al.* (2020) experiments. To further improve marker reliability and eliminate any inbreeding effects, the visible mutation combinations *se^1^ sh^1^*, and independently *se^1^ sp^1^ tt^1^*, were introgressed onto the nearly isogenic (*F *>* *0.99) *D. pseudoobscura* reference genome background to generate 2 independent tester strains for use in outcrossing procedures in experiments identifying recombination events. A full list of *X_SR_* stocks, *X_ST_* inbred lines, the visible mutations they carry, and the National Drosophila Species Stock Center identification for their genetic background is provided in Table 1 and as a reagent table in Supplementary File 1.

**Table 1.** A complete list of lines used.

	Visible markers	Genetic background
*Sex Ratio* (*X_SR_*) segregating stocks		
*Sex Ratio* isolate KBNP2	*y^1^*, *se^1^*, *sh^1^*	14011-0121.06
*Sex Ratio* isolate KBNP2	*se^1^*, *ll^1^*, *sp^1^*, *tt^1^*	14011-0121.08
*Sex Ratio* isolate KBNP2	*se^1^*, *sh^1^*	Lab Line 2020
*Sex Ratio* isolate Z8	*y^1^*, *se^1^*, *sh^1^*	14011-0121.06
*Sex Ratio* isolate Z8	*se^1^*, *ll^1^*, *sp^1^*, *tt^1^*	14011-0121.08
*Sex Ratio* isolate Z8	*se^1^*, *sh^1^*	Lab Line 2020
*Sex Ratio* isolate Z6	*y^1^*, *se^1^*, *sh^1^*	14011-0121.06
*Sex Ratio* isolate Z6	*se^1^*, *ll^1^*, *sp^1^*, *tt^1^*	14011-0121.08
*Sex Ratio* isolate Z6	*se^1^*, *sh^1^*	Lab Line 2020
Standard arrangement (*X_ST_*) inbred lines		
Standard inbred line 1	*y^1^*, *se^1^*, *sh^1^*	14011-0121.06
Standard inbred line 2	*se^1^*, *ll^1^*, *sp^1^*, *tt^1^*	14011-0121.08
Standard inbred line 3	*se^1^*, *sh^1^*	Lab Line 2020
Standard arrangement (*X_ST_*) tester strains		
Standard tester strain 1	*se^1^*, *sh^1^*	14011-0121.94
Standard tester strain 2	*se^1^*, *sp^1^*, *tt^1^*	14011-0121.94

All lines have highly inbred genetic backgrounds with National Drosophila Species Stock Center identifier listed. Inbred lines and tester strains are homozygous for standard arrangement *X* chromosomes. *Sex Ratio* stocks are segregating for the inverted arrangement unmarked *X_SR_* and standard arrangement multiply marked *X_ST_*.

### Recombination experimental design

**Fig. 3.**
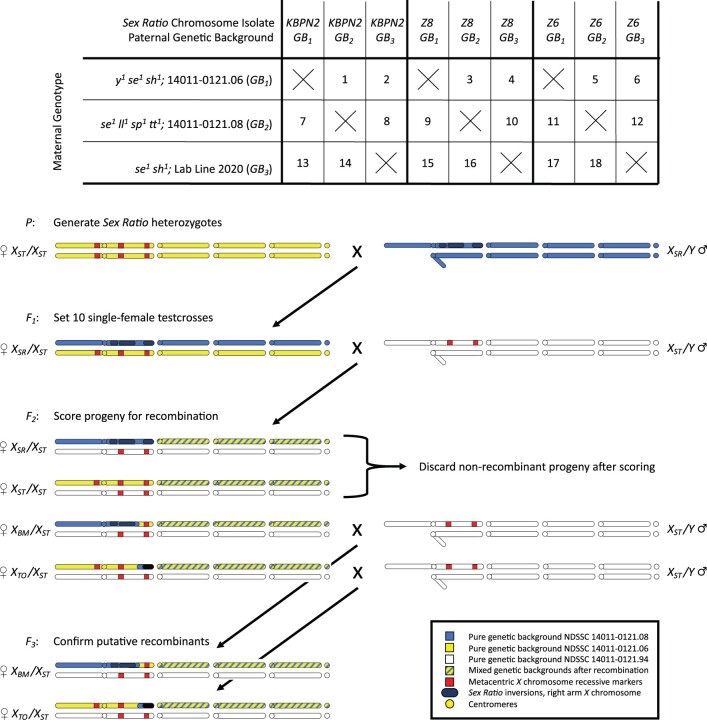
Factorial design matrix and crossing scheme for recombination experiments. The 3×3×3-9 design produced 18 unique *F_1_ X_SR_/X_ST_* genotypes with 9 inbred genotypes excluded. Color-coded crossing scheme shows Cross 1, see Supplementary File 3 for all 18, depicting full *D. pseudoobscura* karyotype consisting of a metacentric *X* chromosome, J-shaped *Y* chromosome, 3 acrocentric autosomes, and 1 pair of dot chromosomes. *F_2_* recombinants are only illustrated as female, but both *F_2_* sexes were scored and backcrossed to confirm recombinants.

To test for recombination in the 6.6-Mb colinear region located between medial and terminal inversions of *D. pseudoobscura X_SR_* chromosomes, a single-block, fully coded, randomized design was conducted with the experimenter blinded to genotypic treatment. The factorial design incorporated 3 independent *X_SR_* chromosome isolates, each on 3 independent autosomal genetic backgrounds, and 3 independent multiply marked *X_ST_* chromosomes on those same 3 autosomal genetic backgrounds $\left(3\times 3\times 3\right)$. Experimental *F_1_* female heterozygotes (*X_SR_/X_ST_*) were generated by performing all pairwise crosses, excluding genotypes that would produce inbred genetic backgrounds $\left(3\times 3\times 3-9\right)$, for a total of 18 unique outbred experimental genotypes (Supplementary Table 1 in Supplementary File 2). Figure 3 provides the factorial experimental design and a color-coded crossing scheme to produce the outbred *F_1_* experimental genotype, the experimental cross itself, and confirmatory testcross of any recombinant *F_2_* progeny (crossing scheme for all 18 unique *F_1_* experimental genotypes is given in Supplementary File 3).

A total of 180 *F_1_* experimental virgin females and 900 tester strain males were collected within 12 h of eclosion over a 3-day period. Females and males were aged in separate vials for an additional 3-day period. Using light CO_2_ anesthesia, 10 females for each genotypic treatment were then individually mated to 5 tester males possessing an independent genetic background (from the *D. pseudoobscura* reference genome) and the respective *X*-linked markers (*se^1^ sh^1^* or alternately *se^1^ sp^1^ tt^1^*). The 180 single-female crosses (18 genotypic treatments with 10 replicates each) were allowed another 3-day CO_2_ recovery period, during which all experimental crosses were coded and randomized. Experimental crosses were maintained at 21°C, 65% relative humidity, and 14:10 h light:dark cycle. The single-female crosses were individually tap transferred onto experimental food for a 3-day egglaying period that occurred when all flies were minimally 1 week and no older than 2 weeks posteclosion. Experimental food was standard cornmeal molasses *Drosophila* media seeded with live yeast. After 3 days, the parental adult was removed under CO_2_ anesthesia and the food was hydrated as needed with 0.5% v/v propionic acid.

Both male and female *F_2_* progeny were scored daily for visible mutations (*se^1^*, *sh^1^* or alternately *se^1^*, *sp^1^*, *tt^1^*) until adult flies stopped emerging, for a total of 17,604 progeny. However, scoring recombination between *se^1^* and *sp^1^ tt^1^* in female progeny was confounded by the presence of *ll^+^* in the tester strain’s colinear region that altered the penetrance of wing vein mutations *sp^1^* and *tt^1^*. Therefore, the 6 genotypic treatments that used the *se^1^*, *sp^1^*, *tt^1^* marker system could not be reliably scored and were excluded from analysis.

When a putative *F_2_* recombinant was identified, this individual was isolated and mated to multiply marked individuals (*se^1^*, *sh^1^*) of the opposite sex to establish a temporary stock. Confirmation that the individual carried a true recombinant *X* chromosome was provided by the transmission and detection of that same marker combination in the subsequent generation. Initial *F_2_* screening identified 102 recombinants among 11,808 progeny scored for visible markers *se^1^* and *sh^1^*. Confirmation tests of these 102 recombinants revealed 10% resulted from errors in scoring the wing vein marker *sh^1^*. Rates of misclassification for *sh^1^* as wild-type *sh^+^* (3/24, exact binomial 95% confidence interval 0.03–0.36) were statistically indistinguishable from the rate of misclassifying *sh^+^* as a mutant *sh^1^* (7/78, exact binomial 95% confidence interval 0.03–0.18).

### Statistical analysis of recombination

An ANOVA with type II sum of squares was conducted testing *X_SR_* chromosome isolate effect and genetic background effect on the observed recombination rate. First, any putative recombinant that was not confirmed in subsequent generations was recoded in the dataset. Second, the recombination rate was calculated as the number of confirmed recombinants divided by the total number of progeny scored for each single-female cross. Third, this proportion was arcsine-transformed ($\theta =\sin^{-1}\sqrt{p}$) to fit ANOVA’s error term normality assumption (Sokal and Rohlf 1995). The linear model analyzed was $\theta_{\mathrm{ijk}}=\gamma_{i}+\tau_{j}+\varepsilon_{\mathrm{ijk}}$; where $\gamma_{i}$ is the effect of the *i*th *X_SR_* chromosome isolate, $\tau_{j}$ is the effect of the *j*th genetic background, and $\varepsilon_{\mathrm{ijk}}$ represents the error term. Because no statistically significant effects related to *X_SR_* chromosome isolate or genetic background were detected, all subsequent analyses did not subdivide the dataset based on these factors.

To investigate whether the observed data were consistent with meiotic crossing-over, the count of recombinants per single-female cross was compared to the Poisson distribution. Assuming meiotic recombination events were rare and occur independently, then the probability of observing a given number of recombinants $\left(x_{i}\right)$ for single-female cross i with $n_{i}$ offspring is $\mathrm{Pr}\left(x_{i};\lambda \right)=\frac{{\left(\lambda n_{i}\right)}^{x_{i}}{\;e}^{-\left(\lambda n_{i}\right)}}{x_{i}!}$, where λ is the experiment-wide rate of recombination. The probability distributions for each single-female cross were combined to generate an experiment-wide distribution accounting for differences in offspring number among crosses. To evaluate the full observed dataset, a $\chi^{2}$ goodness-of-fit test with 4 degrees of freedom was conducted with expected values given by the experiment-wide distribution. The probability of observing a single-female cross with more than 4 recombinants in this design is always less than 0.01. Therefore, the few crosses that produced 5, 6, and 9 recombinants were pooled into a single class consisting of ≥4 recombinants (this is a statistically conservative procedure for this dataset).

To further investigate whether the observed data were consistent with reciprocal-exchange model of meiotic crossing-over, the proportion of recombinants per single-female cross carrying only the terminal inversion (*X_TO_*), and not the basal or medial inversions (*X_BM_*), was compared to the binomial distribution. Assuming recombination events were independent with an equal probability of transmitting either complementary recombinant *X* chromosome to progeny $\left(p=\left(1-q\right)=0.5\right)$, then the probability of observing a given number of recombinants $k_{i}$ for single-female cross i carrying only the terminal inversion is binomially distributed Prki;xi=xikipki1-pxi-ki, where $x_{i}$ is the number of recombinants from cross i with $k_{i}$ total progeny scored. The probability distribution for each single-female cross producing 2 or more recombinants was binned to generate an experiment-wide distribution that controlled for differences in recombinant numbers among crosses. The observed data were then compared to the binned expected distribution with a $\chi^{2}$ goodness-of-fit test with 4 degrees of freedom.

Finally, to investigate whether sex of the progeny scored for recombination had an effect on the chances of observing complementary recombinant *X* chromosomes, a 2×2 contingency table was analyzed. Under a reciprocal-exchange model of meiotic crossing-over with no effect of progeny sex on detection probability, all 4 classes (i.e. females with *X_BM_*, males with *X_BM_*, females with *X_TO_*, and males with *X_TO_*) were expected to occur with equal frequency. The observed data were compared to this expectation (discrete uniform distribution) using a $\chi^{2}$ goodness-of-fit test with 3 degrees of freedom.

### Population genetic modeling

To understand the natural population consequences of the experimentally detected deviations from a reciprocal-exchange meiotic crossing-over, I incorporated recombination into the classical model of *Sex Ratio* evolution (see Appendix A for detailed derivation). Briefly, Edwards (1961) developed a sex-specific, genotypic model for *Sex Ratio* because *X*-linked meiotic drivers cause sustained difference in allele frequencies between the sexes. The extended model in this study used a mating table as a transition matrix to convert the 40 possible mating types (4 male genotypes × 10 female genotypes) to the next generation’s 14 genotypic frequencies (4 male genotypes and 10 female genotypes). The full 40 × 14 mating table is provided as Supplementary File 4 and is rewritten in Appendix A as a system of recursion equations with 14 variables representing the frequency of each genotype. This is a discrete generation model, where the genotypic frequencies in the next generation are a function of the frequencies in the previous generation and parameters *v_i,j_* for relative viability, *f_i,j_* for relative fertility, *k_i_* for transmission in the male germline, and *c_i,j_* for the recombination rate of double heterozygotes in the female germline [Equation (A1) assuming recombination is “meiotic crossing-over” and Equation (A2) assuming recombination is “mitotic unequal exchange”].

I parameterized the extended model of Edwards (1961) with experimental data on viability, fertility, distortion, and recombination (Curtsinger and Feldman 1980; Fuller *et al.* 2020; this study). Making the additional assumption that fitness effects in recombinants were both recessive and proportional to the fraction of 26-Mb *SR* haplotype retained after recombination, experiment-based estimates for all 34 parameters in this extended population genetic model were provided (Table A1). Under these conditions, the equilibrium frequencies of all 14 genotypic variables were solved for by iterating the system of equations with a range of different initial frequency combinations spanning 0.01–0.99. Finally, the modeled equilibrium frequencies assuming recombination occurred as “meiotic crossing-over” or alternately under conditions of “mitotic unequal exchange” were compared to the gold standard of Beckenbach’s (1996) direct cytological observation of *SR* inversion complement for 694 *X* chromosomes sampled from Tucson and Bear Creek, AZ, USA.

## Results

After adjusting for false positives, the observed experiment-wide recombination rate was 0.00779 (92 recombinant chromosomes observed in 11,808 progeny). Substantial heterogeneity among the single-female crosses was observed in recombination rates (for raw counts see Supplementary File 5). The dataset was generated under common garden conditions as a single-block, fully randomized, factorial experimental design with the investigator blinded to treatment, such that the observed heterogeneity was not likely an artifact of design. To investigate the biological sources of recombination rate heterogeneity, and to test the null hypothesis that a reciprocal-exchange model of meiotic crossing-over produced this pattern of heterogeneity, the distribution of observed data was subjected to further statistical analysis.

### No genetic variation for recombination rate

To investigate whether the *X_SR_* chromosome isolate or genetic background affected recombination, a type II ANOVA was conducted on arcsine-transformed rate data. No statistically significant effects were detected for the 3 *X_SR_* chromosome isolates $\left(F_{2,115}=0.57,\;P=0.57\right)$ or the 3 genetic backgrounds $\left(F_{2,115}=0.84,\;P=0.44\right)$ (Table 2). In addition, no statistically significant effects were detected in sex-stratified analysis of the rate data (Supplementary Table 2). Therefore, all subsequent analyses did not subdivide dataset based on *X_SR_* chromosome isolate or genetic background.

**Table 2.** Recombination rate ANOVA table.

Source of variation	*df*	SS	MS	*F_s_*	*P*-Value
*X_SR_* chromosome isolate	2	19.95	9.98	0.57	0.57
Genetic background	2	29.16	14.58	0.84	0.44
Residuals	115	2,005.82	17.44		
Total	119	2,054.93			

No genetic variation for recombination rate was detected when analyzing the effect of 3 *X_SR_* chromosome isolates and 3 heterozygous genetic backgrounds with type II ANOVA.

### Recombination events were clustered among single-female crosses

**Fig. 4.**
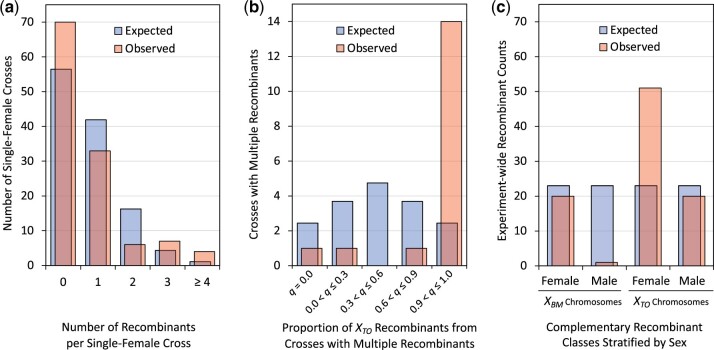
Observed versus expected distribution of recombination events. a) Observed recombination events (red) were clustered among single-female crosses when compared with Poisson expectations (blue) based on experiment-wide recombination rate $\left(\chi_{\left[4\right]}^{\;2}=21.03,\;P=1.43\times 10^{-4}\right)$ (see Table 3). b) Proportion of recombinants $\left(q\right)$ carrying *X_TO_* were clustered within single-female crosses when compared with expected proportions from the binomial distribution. Multiple recombinants from single-female crosses (red) were more likely to all be *X_TO_* than expected by chance (blue) $\left(\chi_{\left[4\right]}^{\;2}=64.30,\;P=1.75\times 10^{-13}\right)$ (see Table 4). c) Recovery rates of complementary recombinants were neither equivalent between nor independent of *F_2_* progeny sex. The observed data (red) differed from uniform discrete expectations (blue) with statistical significance $\left(\chi_{\left[3\right]}^{\;2}=55.9,\;P=2.15\times 10^{-12}\right)$ (see Table 5). A deficit of both complementary recombinants (*X_TO_* and *X_BM_*) was detected in the hemizygous (male) state and was particularly severe for *X_BM_*.

To investigate whether the data were consistent with meiotic crossing-over (i.e. independence of recombination events among single-female crosses), the observed count of recombinants per single-female cross was compared to the Poisson distribution using the experiment-wide recombinant frequency as the rate parameter λ (Fig. 4a). A $\chi^{2}$ goodness-of-fit test with 4 degrees of freedom revealed that the observed recombination events were not independent $\left(\chi_{\left[4\right]}^{\;2}=21.02,\;P=1.43\times 10^{-4}\right)$ (Table 3). Substantially more single-female crosses produced either no recombinants or multiple recombinants than were expected assuming independence of recombination events. The coefficient of dispersion $\left(D=\frac{\sigma^{2}}{\mu }\right)$ differed with statistical significance from the Poisson distribution’s expected value of 1 $\left(\chi_{\left[119\right]}^{\;2}=296.70,\;P=1.09\times 10^{-17}\right)$, indicating that the data were strongly clustered. The same pattern of overdispersion (clustering) was present in sex-stratified analysis of observed data (Supplementary Table 3). Strong clustering of observed data rejects the null hypothesis that reciprocal exchange in meiosis produced this dataset and suggests that multiple recombinant *X* chromosomes recovered from the same experimental *F_1_* single-female cross were clonal.

**Table 3.** Recombination events were not independent among *F_1_* single-female crosses.

Category	Expected	Observed	(Obs − Exp)^2^/Exp
0 recombinants	56.44	70	3.26
1 recombinant	41.87	33	1.88
2 recombinants	16.24	6	6.46
3 recombinants	4.36	7	1.60
≥4 recombinants	1.09	4	7.83

			χ^2^ = 21.03

The number of recombination events per single-female cross, if events were independent, should be Poisson distributed. A $\chi^{2}$ goodness-of-fit test revealed the observed recombination events per single-female cross differed with statistically significance from Poisson expectations using an experiment-wide recombination rate $\left(\chi_{\left[4\right]}^{\;2}=21.03,\;P=1.43\times 10^{-4}\right)$. Clustering was even more extreme than the $\chi^{2}$ test indicates due to conservative procedure of binning crosses producing 4 or more recombinants (see Fig. 4a).

### Multiple recombination events were clustered within single-female crosses

To investigate whether the observed data were consistent with meiotic crossing-over (i.e. independence of recombination events within a single-female cross), the proportion of recombinants $\left(q\right)$ carrying *X_TO_* within the progeny from a single-female cross was compared to the binomial distribution (Fig. 4b). A $\chi^{2}$ goodness-of-fit test revealed multiple recombinants from a single-female cross differed with statistical significance from binned expectations assuming independence $\left(\chi_{\left[4\right]}^{\;2}=64.30,\;P=1.75\times 10^{-13}\right)$ (Table 4). The same pattern of clustering was present in sex-stratified analysis of data, indicating that hemizygous fitness effects were not solely responsible for this deviation from independence (Supplementary Table 4). Multiple recombinants from a given single-female cross were more likely to carry the same inversion complement than expected by chance, again rejecting the null hypothesis of meiotic reciprocal exchange and further suggesting that multiple recombinant *X* chromosomes recovered in from the same experimental *F_1_* single-female cross were clonal.

**Table 4.** Multiple recombination events were not independent within *F_1_* single-female crosses.

Category	Expected	Observed	(Obs − Exp)^2^/Exp
*q *=* *0.0	2.44	1	0.85
0.0 < *q *≤* *0.3	3.69	1	1.96
0.3 < *q *≤* *0.6	4.74	0	4.74
0.6 < *q *≤* *0.9	3.69	1	1.96
*q *=* *1.0	2.44	14	54.79

			χ^2^ = 64.30

Proportion of recombinants $\left(q\right)$ carrying one inversion complement (*X_TO_*) in single-female crosses producing multiple recombinants *via* meiotic crossover should be binomially distributed. A $\chi^{2}$ goodness-of-fit test revealed statistically significant deviation from these expectations $\left(\chi_{\left[4\right]}^{\;2}=64.30,\;P=1.75\times 10^{-13}\right)$. Multiple recombinants from single-female crosses (predominantly *X_TO_*) were much more likely to carry the same inversion complement than expected by chance (see Fig. 4b).

### Recovery rates of recombinants were neither equivalent nor independent of sex

To investigate whether the sex of progeny scored for recombination had an effect on the probability of observing a recombinant (i.e. recombination events in *F_1_* female germline were independent of *F_2_* progeny sex), the observed data were compared to a discrete uniform distribution (Fig. 4c). The observed data differed from expectations with statistical significance $\left(\chi_{\left[3\right]}^{\;2}=55.9,\;P=2.15\times 10^{-12}\right)$ (Table 5). These results demonstrated that recovery of recombinant *X* chromosomes was strongly dependent on the *F_2_* sex in which they were detected, with the most noticeable deficit occurring in the male *X_BM_* class.

**Table 5.** Recovery rates of complementary recombinants were not equivalent among *F_2_* progeny sex.

Category	Expected	Observed	(Obs − Exp)^2^/Exp
*X_BM_*, female	23	20	0.39
*X_BM_*, male	23	1	21.04
*X_TO_*, female	23	51	34.09
*X_TO_*, male	23	20	0.39

			χ^2^ = 55.91

The observed recombinants classified by sex and inversion complement differed from uniform discrete expectations with statistical significance $\left(\chi_{\left[3\right]}^{\;2}=55.9,\;P=2.15\times 10^{-12}\right)$ (see Fig. 4c). Observation of complementary recombinants was strongly dependent on the *F_2_* sex in which they were detected, with the most noticeable deficit occurring in the male *X_BM_* class.

The observed recovery rates for *X_BM_* recombinants in both males and females were much lower when compared to the complementary recombinant *X_TO_*. $\chi^{2}\;$tests of independence failed even when different rates of production were estimated from the data for complementary recombinant classes $\left(\chi_{\left[2\right]}^{\;2}=34.7,\;P=1.06\times 10^{-7}\right)$ (Supplementary Table 5). There was also a deficit of both complementary recombinants (*X_TO_* and *X_BM_*) detected in the hemizygous (male) state compared to the protected heterozygous (female) state. Again, $\chi^{2}\;$tests of independence failed after different rates for both sexes and complementary classes were estimated from the data $\left(\chi_{\left[1\right]}^{\;2}=5.04,\;P=0.0143\right)$ (Supplementary Table 5). Even after these corrections, the most noticeable deficit was still due to the hemizygous (male) *X_BM_* class, a genotype with notably reduced viability in the laboratory (Wallace 1948; Fuller *et al.* 2020). The process of recombination itself causing asymmetric recessive viability defects is consistent with a model of mitotic exchange generating a chromosomal deletion for *X_BM_*, and a complementary duplication for *X_TO_*, via unequal crossing-over in the female GSC.

### Experimental results predict recombinant rarity in the wild

The natural population consequences of these experimental results are not intuitive; therefore, I explored their effect with the classical model of *Sex Ratio* evolution extended to include recombination (Edwards 1961). This is a sex-specific, genotypic model for *Sex Ratio* with viability, fertility, and transmission parameters drawn from published experimental data (Curtsinger and Feldman 1980; Fuller *et al.* 2020). Evolution of all 14 genotypes formed from *X_ST_*, *X_SR_*, *X_BM_*, and *X_TO_* chromosomes was investigated by iterating a system of equations generated from the full 40 × 14 mating table provided in Supplementary File 4 (see Appendix A for detailed derivation and system of equations).

First, “meiotic crossing-over” conditions were investigated by assuming *X_SR_/X_ST_* recombination was due to meiotic crossing-over with an experiment-wide recombination rate ${(c}_{1,2}=0.00779)$ while making the additional assumption that fitness effects in recombinants are both recessive and proportional to the fraction of 26-Mb *SR* haplotype retained after recombination (Table A1). The equilibrium frequencies of all 14 genotypes, with a range of initial frequency combinations between 0.01 and 0.99, all converged on values reported in Table 6 (column “meiotic crossing-over”).

**Table 6.** Numerical solutions for equilibrium genotypic frequencies in *D. pseudoobscura Sex Ratio* system.

Genotype	Frequency notation	Meiotic crossing-over	Unequal mitotic exchange
*X_ST_Y*	*x_1_*	3.5 × 10^−1^	3.5 × 10^−1^
*X_SR_Y*	*x_2_*	3.6 × 10^−2^	4.6 × 10^−2^
*X_BM_Y*	*x_3_*	1.6 × 10^−2^	**4.1 × 10^−5^**
*X_TO_Y*	*x_4_*	2.7 × 10^−3^	1.2 × 10^−3^
*X_ST_X_ST_*	*x_1,1_*	4.1 × 10^−1^	4.3 × 10^−1^
*X_ST_X_SR_*	*x_1,2_*	1.2 × 10^−1^	1.6 × 10^−1^
*X_ST_X_BM_*	*x_1,3_*	4.8 × 10^−2^	**9.5 × 10^−4^**
*X_ST_X_TO_*	*x_1,4_*	6.4 × 10^−3^	5.0 × 10^−3^
*X_SR_X_SR_*	*x_2,2_*	5.8 × 10^−3^	9.8 × 10^−3^
*X_SR_X_BM_*	*x_2,3_*	5.3 × 10^−3^	**1.6 × 10^−4^**
*X_SR_X_TO_*	*x_2,4_*	8.4 × 10^−4^	8.8 × 10^−4^
*X_BM_X_BM_*	*x_3,3_*	1.1 × 10^−3^	**1.1 × 10^−7^**
*X_BM_X_TO_*	*x_3,4_*	3.8 × 10^−4^	**5.7 × 10^−6^**
*X_TO_X_TO_*	*x_4,4_*	2.2 × 10^−5^	1.0 × 10^−5^

The equilibrium frequencies are listed for all genotypes (4 male hemizygous states, 4 female homozygous states, and 6 female heterozygous states) in the recombination extensions of Edwards (1961) model of *Sex Ratio* evolution. Full system of 14 equations is derived in Appendix A by assuming recombination was either reciprocal exchange caused by meiotic crossing-over in the primary oocyte or alternately caused by unequal mitotic exchange in female GSCs. Under alternate assumptions, the largest differences all involve the genotypes with *X_BM_* and are highlighted in bold.

**Fig. 5.**
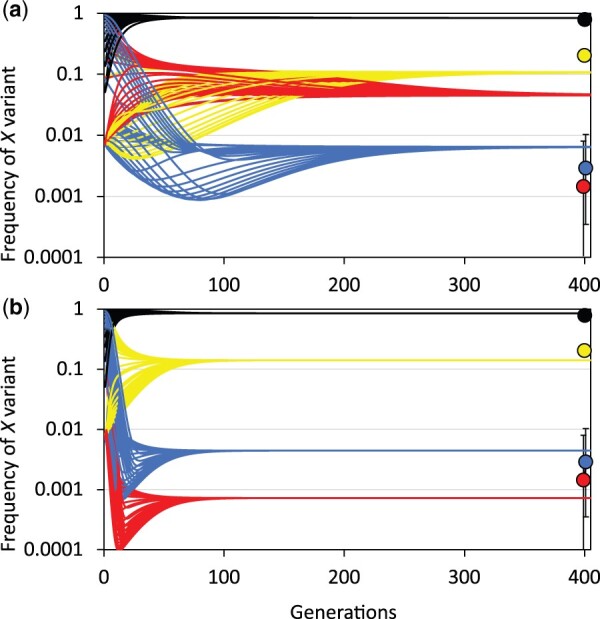
Modeled evolutionary trajectories for *X* chromosome variants in *D. pseudoobscura*. Change in population frequency of *X_ST_* (black), *X_SR_* (yellow), *X_BM_* (red), and *X_TO_* (blue) chromosomes with initial frequencies ranging from 0.01 to 0.99. For comparison, Beckenbach’s (1996) observations from AZ, USA are given as color-coded points on the far right with exact binomial 95% confidence intervals. a) Extension of Edwards (1961) population genetic model assuming recombination is reciprocal-exchange “meiotic crossing-over.” b) The same model incorporating experimentally determined asymmetries in recombination due to “unequal mitotic exchange.” The Edwards (1961) model incorporating unequal mitotic exchange is a much better fit to the frequencies observed in the wild (see Table 7).

Summing across genotypes, the modeled equilibrium frequencies of recombinant *X* chromosomes under “meiotic crossing-over” conditions were 0.045 (*X_BM_*) and 0.0065 (*X_TO_*), with *SR* inversion linkage disequilibrium $r^{2}=0.62$ (Fig. 5a and Table 7). These results can be evaluated by comparison to the gold standard of Beckenbach’s (1996) direct cytological observation of *SR* inversion complement for 694 *X* chromosomes sampled from Tucson and Bear Creek, AZ, USA. The *SR* population genetic model assuming “meiotic crossing-over” conditions when compared to direct natural population observations reveals: (1) incorrect prediction of the rank order of recombinant *X* chromosomes, (2) inaccurate estimates of the natural population *X_SR_*, *X_BM_*, and *X_TO_* frequencies, and (3) a large discrepancy in linkage disequilibrium of *SR* inversions (Table 7).

**Table 7.** Observed and modeled *D. pseudoobscura X* chromosome variant frequencies and linkage disequilibrium of *SR* haplotype inversions.

	Direct cytological observations	Decay model of inversion LD	*SR* model assuming meiotic crossover	*SR* model with unequal mitotic exchange
*X_ST_*	0.79	0.64	0.84	0.85
*X_SR_*	0.20	0.04	0.11	0.14
*X_BM_*	0.001	0.16	0.045	0.001
*X_TO_*	0.003	0.16	0.007	0.004
*r^2^*	0.97	0.00	0.62	0.97

Direct cytological observations of Beckenbach (1996) were compared to the modeled frequencies and linkage disequilibrium from the decay model of Fuller *et al.* (2020) as well as the recombination extensions of Edwards (1961) derived in Appendix A. The best fit to natural population frequencies of *X_ST_* and *X_SR_* chromosomes, their recombinants *X_BM_* and *X_TO_*, and linkage disequilibrium (*r^2^*) of *SR* haplotype inversions was the model for *Sex Ratio* evolution incorporating unequal mitotic exchange through experimentally determined asymmetries in recombination.

Second, “mitotic unequal exchange” conditions were investigated where *X_SR_/X_ST_* recombination was modeled by calculating independent complementary recombinant class-specific rates ($c_{3}$ and $c_{4}$) from experimental data in the protected heterozygous state (i.e. from *F_2_* female progeny only in Fig. 4c) $\left(c_{3}=0.0072,\;c_{4}=0.018\right)$. Similarly, hemizygous relative viabilities for the complementary recombinants were calculated as the experimentally observed proportional deficit of recombinants observed in *F_2_* male versus *F_2_* female progeny in Fig. 4c$\left(v_{3}=0.05,\;v_{4}=0.39\right)$. All other parameters remained identical to the “meiotic crossing-over” conditions. Solving the new system of equations [Equation (A2)], with new parameter values based experimentally observed asymmetries in recombination, revealed that the largest changes in equilibrium frequencies all involve genotypes containing *X_BM_* (bold values in Table 6).

Incorporating the experimentally observed *X_SR_/X_ST_* recombination asymmetries in this manner, and summing across genotypes, the modified model described above produced equilibrium frequencies of 0.00072 (*X_BM_*) and 0.0044 (*X_TO_*), both of which are within the 95% confidence intervals of Beckenbach’s (1996) sampled frequencies (Fig. 5b). Further benchmarking against Beckenbach (1996) demonstrated that the *SR* population genetic model under “unequal mitotic exchange” conditions could: (1) correctly predict rank order of recombinant *X* chromosome frequencies, (2) improve fit to natural population *X_SR_*, *X_BM_*, and *X_TO_* frequencies, and (3) closely match *SR* inversion linkage disequilibrium in the wild (Table 7). The close match of *D. pseudoobscura* natural population observations and modeled equilibrium frequencies of *X_SR_*, *X_BM_*, and *X_TO_* chromosomes under the “unequal mitotic exchange” conditions both explains the rarity of *SR* recombinants in the wild and nullifies our previous conclusion that epistatic selection must act to maintain the 26-Mb *SR* haplotype (Fuller *et al.* 2020).

## Discussion

The present results demonstrate that recombination events located between nonoverlapping inversions of *Sex Ratio* chromosome heterozygotes in *D. pseudoobscura* were not as rare as previously estimated. Importantly, the recombination events exhibited strong clustering both among and within single-female crosses. The hypothesis that canonical meiotic crossing-over (reciprocal exchange) generated this recombination dataset was unambiguously rejected (Fig. 4). At least 17 of 50 single-female crosses that produced recombinants can be statistically attributed to mitotic exchange in female GSCs or an earlier stage in development (Fig. 2). As a result, at least 64% of the total recombinant progeny observed in this experiment (59/92) originated from female germline mitotic exchange. The remaining 36% of recombinants occurred in single-female crosses that produced only one recombinant in the 3-day egglaying period and therefore provided insufficient information to determine whether their origin was due to mitotic exchange or meiotic crossing-over. Thus, while 92 recombinant chromosomes were observed in 11,808 progeny scored, these 92 recombinants may be the product of as few as 50 unique recombination events.

The observed experiment-wide recombination rate of 92 recombinant chromosomes per 11,808 progeny scored (0.00779) was within the expected range if a single *X_SR_/X_ST_* heterozygous GSC was converted via mitotic exchange per single-female cross (expected range 0.00417–0.00781, calculated assuming 2–3 GSCs per ovariole, 16–20 ovarioles per ovary, 2 ovaries per female). In principle, mitotic exchange may occur after asymmetric division of GSCs in the cystoblast; but because each cyst only yields one oocyte, any mitotic exchanges subsequent to differentiation from GSCs cannot be the source of clustering (Fig. 2). Conversely, the most extreme single-female recombination rates observed in this experiment (e.g. 6/47 and 9/122) may be caused by mitotic exchange in primordial germ cells prior to their development into GSCs, therefore leading to a subpopulation of clonal GSCs with the converted genotype. Based on the close match with the expected range, the large majority of the observed recombination events appear to have occurred as mitotic exchange in female GSCs rather than in an earlier stage.

The statistical approach of this study cannot provide direct evidence of unequal mitotic exchange, but asymmetric patterns in the dataset (i.e. specific deficit of *X_BM_* recombinants or deficit of hemizygous recombinants more generally) were consistent with unequal exchange. The most parsimonious explanation for the different recovery rates of complementary recombinants, and the observed recombination-induced recessive viability defects, is unequal exchange generating a chromosomal deletion for *X_BM_* and a complementary duplication for *X_TO_*. However, it is possible that more complex chromosomal aberrations stemming from the mitotic exchange may have caused the disproportionate deficit of hemizygous *X_BM_* recombinants observed in Fig. 4c. In future studies, identification of recombination breakpoints with long-read sequencing could provide direct evidence for this scenario’s predictions that: (1) clustered recombinants had clonal origins, (2) repetitive genetic elements mediated ectopic exchange, and (3) deleted and/or duplicated regions at breakpoints caused the observed fitness defects. Sequencing-based insights into mechanism may also help establish if the recombination rate represents a normal baseline of mitotic exchange in the genome or is due to unique structural features of the 6.6-Mb colinear region of *X_SR_* chromosomes.

Mitotic exchange in *Drosophila* female GSC is not entirely unknown but remains understudied because, under normal circumstances, its signal is swamped by the noise of meiotic crossing-over (Ashburner *et al.* 2004). Indeed, the previous evidence of GSC mitotic exchange comes from extreme conditions such as X-ray irradiation or compound *X* and *Y* chromosomes in the female germline (Neuhaus 1936; Bateman and Chandley 1965). However, if low levels of female GSC mitotic exchange are universal, then it is interesting to speculate that they are (at least in part) responsible for the unpatterned residual recombination observed in mutants of *Drosophilamelanogaster*’s minichromosome maintenance complex (*mei-MCM*) that regulates meiotic crossover formation (Liu *et al.* 2000; Kohl *et al.* 2012; Hartmann *et al.* 2019).

Extending this same logic, mitotic exchange in the female germline provides a convenient explanation for the counter-intuitive results implicating *Bloom syndrome helicase* (*Blm*) as an anticrossover factor in mitosis but pro-crossover factor in meiosis (Hatkevich and Sekelsky 2017). *Blm* mutants elevate mitotic exchange due to failed synthesis-dependent strand annealing and double-Holliday junction dissolution when repairing double-strand breaks that occur outside of meiosis (Adams *et al.* 2003; McVey *et al.* 2007). *Blm* mutants also exhibit unpatterned, *mei-9*-independent, large residual “meiotic” recombination (∼70% of wild-type rates compared to ∼10% residual recombination of *mei-MCM* mutants) (Hatkevich *et al.* 2017). Rather than postulating 2 different functions of *Blm*, it is possible that the high residual recombination rate assumed to be “meiotic” was instead just a compounded product of elevated exchange in the mitotic divisions of the female germline. Tests of this and other mitotic exchange hypotheses can be conducted as sensitized assay with meiotic mutants (Zwick *et al.* 1999), does not require sequence-based analysis, and can be readily disproven with statistical analysis of clustering in single-female crosses.

### Modeling recombinant rarity in natural populations

Returning to the specific case of *Sex Ratio* system in *D. pseudoobscura*, the higher recombination rate observed in this study intuitively suggests that recombinant *Sex Ratio* chromosomes should be more frequent in the wild (greater than 10% of *X* chromosome variants at equilibrium). However, both *X_TO_* and *X_BM_* are still quite rare (less than 1%) in natural population samples with only 5 instances known from greater than 10,000 *X* chromosomes tested (Sturtevant and Dobzhansky 1936; Wallace 1948; Beckenbach 1996; Fuller *et al.* 2020). To investigate whether the properties of unequal mitotic exchange could explain the low frequencies of *X_TO_* and *X_BM_* in the wild, it was necessary to introduce recombination into the Edwards (1961) population genetic model of *Sex Ratio* in *D. pseudoobscura* (see Appendix A for model extension).

The extended model of *Sex Ratio* evolution in *D. pseudoobscura* was explored under 2 recombination scenarios “meiotic crossing-over” and “unequal mitotic exchange.” Comparison of Fig. 5a and b and examination of Table 7 reveal that the “unequal mitotic exchange” model was a better predictor of both natural population frequencies and the observed pattern of *SR* inversion linkage disequilibrium. Importantly, the alternative scenario of “unequal mitotic exchange” simply replaces the standard assumption that all recombination events are (1) independent, (2) reciprocal exchange, and (3) do not directly cause deleterious fitness effects, with parameter values reflecting the experimentally observed recombination asymmetries. Thus, the “unequal mitotic exchange” conditions do not rely on any specific theoretical model of recombination, and the use of this nomenclature is based the parsimonious explanation of the data and used as a convenient short-hand description to differentiate these conditions from the standard reciprocal-exchange model of “meiotic crossing-over.”

Whereas the statistical analysis of the experimental data strongly rejects the null hypothesis that “meiotic crossing-over” generated the recombination dataset (Fig. 4), it is the modeling of natural population frequencies that reveals adopting the alternative hypothesis of “unequal mitotic exchange” is important for understanding the evolutionary dynamics of the *D. pseudoobscura SR* system (Fig. 5). Of the 3 main experimental results, (1) higher recombination rates, (2) clustered recombination events, and (3) asymmetric recovery of complementary recombinant classes, it appears that the last of these has the greatest importance in explaining the equilibrium frequencies of *X* chromosome variants in natural populations. This analysis suggests that incorporating the experimentally determined deficit of hemizygous recombinants into the population model in the form of recombination-induce viability defects is sufficient to explain *X_BM_* and *X_TO_* rarity in the wild, thereby eliminating the need to invoke additional epistatic selection to maintain the highly differentiated 26-Mb *SR* haplotype.

### Implications for meiotic drive and mitotic drive

The causal mechanism for *D. pseudoobscura Sex Ratio* segregation distortion, and the underlying genetic basis, remains unknown. The results of this study have major implications for the frequency of recombinant *Sex Ratio* chromosomes in natural populations, and therefore, the population genetic inference on historical selection regimes maintaining the highly differentiated 26-Mb *SR* haplotype. However, the experimental results presented here do not alter our current understanding of the genetic architecture of the *Sex Ratio* segregation distortion phenotype. Thus, the model in Fuller *et al.* (2020) of: (1) a causal, but weak, distortion locus residing in the proximal half of the right arm of the *X_SR_* chromosome and (2) a modifier locus in the distal half of the same chromosome arm that strengthens distortion to nearly 100%, but is incapable of distorting on its own, is not altered by this study.

In contrast, the discovery that female germline mitotic exchange is the major source of *Sex Ratio* chromosome recombination in *D. pseudoobscura* does potentially have implications for mitotic drive. Mitotic drive, as a population genetic mechanism, describes the outcome of competition among lineages in GSCs where asymmetric division and self-renewal can lead to differential transmission to the next generation. For mitotic drive to impact natural population allele frequencies requires: (1) genetic differentiation among lineages in the germline and (2) that genetic differentiation must confer a systematic proliferative advantage (via increased division, increased longevity, or some other mechanism). Interestingly, mitotic drive has been demonstrated in *D. melanogaster* where induced *chinmo* mutant lineages selectively evict nonmutant lineages by remodeling the extra cellular matrix in the male GSC niche (Tseng *et al.* 2022). While mitotic exchange in *X_SR_/X_ST_* heterozygotes of *D. pseudoobscura* did generate new genetic lineages in GSCs (fulfilling the first condition) and there was greater overall representation of *X_TO_* than *X_BM_* in the progeny (the pattern of mitotic drive), there is no evidence to suggest that this result was due to a proliferative advantage of *X_TO_* in the GSC niche of *F_1_* experimental females. Instead, I have favored the simpler explanation that unequal exchange causes reduced *F_2_* viabilities that are reflected in the asymmetric patterns in the dataset (i.e. specific deficit of *X_BM_* recombinants and deficit of hemizygous recombinants more generally).

### Conclusion

The recombination rate in the 6.6-Mb colinear region of *D. pseudoobscura X_SR_/X_ST_* heterozygotes was revised upward to 0.00779 (92 recombinant chromosomes in 11,808 progeny) using an improved experimental design. Statistical analysis of rate heterogeneity revealed that the majority of these recombination events did not occur as independent meiotic crossover events in primary oocytes, but rather they were clustered in a manner consistent with unequal mitotic exchange in the female GSCs. Incorporating the experimentally observed asymmetries in recombination into a sex-specific, genotypic models of *Sex Ratio* evolution provided a substantially better fit to natural population frequencies and thereby eliminates the need to invoke any additional selection to maintain the 26-Mb *SR* haplotype in *D. pseudoobscura*.

## Supplementary Material

jkac264_Supplementary_File_S1

jkac264_Supplementary_File_S2

jkac264_Supplementary_File_S3

jkac264_Supplementary_File_S4

jkac264_Supplementary_File_S5

## Data Availability

The data underlying this article are available in the article and in its online supplementary material. *D. pseudoobscura* stocks listed in Table 1 and further described in reagent table in Supplementary File 1 are available upon request. The author affirms that all data necessary for confirming the conclusions of the article are present within the article, figures, and tables. Raw counts from the recombination experiments are provided in Supplementary File 5. Supplemental material is available at G3 online.

## Appendix A: Population genetic modeling

To determine the implications of the experimental results for natural populations, the Edwards (1961) model of *Sex Ratio* (*SR*) in *Drosophila pseudoobscura* was extended to include recombination. Following the original model, female genotypes for standard arrangement *X* chromosomes and the multiply inverted *SR* chromosome variants are *X_ST_/X_ST_*, *X_ST_/X_SR_*, and *X_SR_/X_SR_* with frequencies denoted by $x_{1,1}$, $x_{1,2}$, and $x_{2,2}$, respectively. The male genotypes are *X_ST_/Y* and *X_SR_/Y* with respective frequencies $x_{1}$ and $x_{2}$, such that $x_{1}+x_{2}+x_{1,1}+x_{1,2}+x_{2,2}=1$. Let the relative viabilities of these 5 genotypes be $v_{1}$, $v_{2}$, $v_{1,1}$, $v_{1,2}$, $v_{2,2}$, with relative fertilities $f_{1}$, $f_{2}$, $f_{1,1}$, $f_{1,2}$, $f_{2,2},\;$where the subscript denotes the corresponding genotype. To model *X*-linked segregation distortion, male genotypes produce a proportion of female offspring $k_{i}$, with $k_{1}=0.5$ representing mendelian transmission of *X_ST_* chromosomes and $k_{2}=1.0$ representing complete distortion of *X_SR_* chromosomes.

To incorporate recombination into the Edwards (1961) model, let exchange in the 6.6-Mb colinear region located between medial and terminal inversions of *SR* chromosomes occur with frequency $c_{1,2}$ in the double heterozygotes *X_SR_/X_ST_*. Exchange in the colinear region generates 2 new recombinant *X* chromosomes: *X_BM_*, with basal and medial inversions and approximately two-thirds of the 26-Mb *Sex Ratio* haplotype, and *X_TO_*, which carries the terminal inversion only and about one-third of the 26-Mb *Sex Ratio* haplotype. Male genotypic frequency of *X_BM_/Y* and *X_TO_/Y* is denoted $x_{3}$ and $x_{4}$, respectively, with corresponding relative viabilities and relative fertilities of $v_{3}$, $v_{4}$ and $f_{3}$, $f_{4}$. Following this notation, the female genotypes *X_ST_/X_BM_*, *X_ST_/X_TO_*, *X_SR_/X_BM_*, *X_SR_/X_TO_*, *X_BM_/X_BM_*, *X_BM_/X_TO_*, and *X_TO_/X_TO_* have frequencies $x_{1,3}$, $x_{1,4}$, $x_{2,3}$, $x_{3,3}$, $x_{3,4}$, $x_{4,4}$ and corresponding relative viabilities $v_{1,3}$, $v_{1,4}$, $v_{2,3}$, $v_{3,3}$, $v_{3,4}$, $v_{4,4}$ and relative fertilities $f_{1,3}$, $f_{1,4}$, $f_{2,3}$, $f_{3,3}$, $f_{3,4}$, $f_{4,4}$. All genotypes, genotypic frequencies, and their corresponding parameters (relative viability, relative fertility, male germline *X* chromosome transmission, and female germline *X* chromosome recombination) are given in the mating table provided in Supplementary File 4.

To calculate genotypic frequencies in the next generation, a mating table was created that lists all genotypic frequencies of offspring produced from all 40 mating types (4 possible male genotypes × 10 possible female genotypes). The full table (40 parental mating types generating 14 offspring genotypes) is given in Supplementary File 4. After correcting for the frequency and fertility of each parental mating type, as well as relative viability of each offspring class, the genotypic frequencies of reproductively mature adults in the next generation are given by system of equations:

(A1) $$\begin{array}{l} \left(1\right)\;Tx_{1}^{\prime }=\frac{v_{1}}{2}\left({f_{1}x}_{1}\left({1-k}_{1}\right)+f_{2}x_{2}\left({1-k}_{2}\right)+f_{3}x_{3}\left({1-k}_{3}\right)+f_{4}x_{4}\left({1-k}_{4}\right)\right)\\ \;\;\;\;\;\;\;\;\;\;\;\;\;\;\;\;\;\times \left(f_{1,3}x_{1,3}+f_{1,4}x_{1,4}+{2f_{1,1}x}_{1,1}+{c_{3,4}f_{3,4}x}_{3,4}+{\left(1-c_{1,2}\right)f_{1,2}x}_{1,2}\right)\\ \left(2\right)\;Tx_{2}^{\prime }=\frac{v_{2}}{2}\left(f_{1}x_{1}\left({1-k}_{1}\right)+{f_{2}x}_{2}\left({1-k}_{2}\right)+{f_{3}x}_{3}\left({1-k}_{3}\right)+{f_{4}x}_{4}\left({1-k}_{4}\right)\right)\\ \;\;\;\;\;\;\;\;\;\;\;\;\;\;\;\;\;\times \left(f_{2,3}x_{2,3}+f_{2,4}x_{2,4}+{2f_{2,2}x}_{2,2}+{c_{3,4}f_{3,4}x}_{3,4}+{\left(1-c_{1,2}\right)f_{1,2}x}_{1,2}\right)\\ \begin{array}{l} \left(3\right)\;Tx_{3}^{\prime }=\frac{v_{3}}{2}\left(f_{1}x_{1}\left({1-k}_{1}\right)+f_{2}x_{2}\left({1-k}_{2}\right)+f_{3}x_{3}\left({1-k}_{3}\right)+f_{4}x_{4}\left({1-k}_{4}\right)\right)\\ \;\;\;\;\;\;\;\;\;\;\;\;\;\;\;\;\;\times \left(f_{1,3}x_{1,3}+f_{2,3}x_{2,3}+{2f_{3,3}x}_{3,3}+{c_{1,2}f_{1,2}x}_{1,2}+{\left(1-c_{3,4}\right)f_{3,4}x}_{3,4}\right)\\ \left(4\right)\;Tx_{4}^{\prime }=\frac{v_{4}}{2}\left(f_{1}x_{1}\left({1-k}_{1}\right)+f_{2}x_{2}\left({1-k}_{2}\right)+f_{3}x_{3}\left({1-k}_{3}\right)+f_{4}x_{4}\left({1-k}_{4}\right)\right)\\ \;\;\;\;\;\;\;\;\;\;\;\;\;\;\;\;\;\times \left(f_{1,4}x_{1,4}+f_{2,4}x_{2,4}+{2f_{4,4}x}_{4,4}+{c_{1,2}f_{1,2}x}_{1,2}+{\left(1-c_{3,4}\right)f_{3,4}x}_{3,4}\right)\\ \begin{array}{l} \left(5\right)\;Tx_{1,1}^{\prime }=\frac{1}{2}\left(f_{1}k_{1}x_{1}v_{1,1}\right)\left({f_{1,3}x}_{1,3}+{f_{1,4}x}_{1,4}+{2f_{1,1}x}_{1,1}+c_{3,4}{f_{3,4}x}_{3,4}+\left(1-c_{1,2}\right)f_{1,2}x_{1,2}\right)\\ \left(6\right)\;Tx_{1,2}^{\prime }=\frac{1}{2}\left({f_{1}k}_{1}x_{1}v_{1,2}\right)\left(f_{2,3}x_{2,3}+{f_{2,4}x}_{2,4}+{2f_{2,2}x}_{2,2}+c_{3,4}{f_{3,4}x}_{3,4}+\left(1-c_{1,2}\right)f_{1,2}x_{1,2}\right)\\ \;\;\;\;\;\;\;\;\;\;\;\;\;\;\;\;\;+\frac{1}{2}\left(f_{2}k_{2}x_{2}v_{1,2}\right)\left(f_{1,3}x_{1,3}+{f_{1,4}x}_{1,4}+{2f_{1,1}x}_{1,1}+c_{3,4}{f_{3,4}x}_{3,4}+\left(1-c_{1,2}\right)f_{1,2}x_{1,2}\right)\\ \begin{array}{l} \left(7\right)\;Tx_{1,3}^{\prime }=\frac{1}{2}\left({f_{1}k}_{1}x_{1}v_{1,3}\right)\left(f_{1,3}x_{1,3}+{f_{2,3}x}_{2,3}+{2f_{3,3}x}_{3,3}+c_{1,2}{f_{1,2}x}_{1,2}+\left(1-c_{3,4}\right){f_{3,4}x}_{3,4}\right)\\ \;\;\;\;\;\;\;\;\;\;\;\;\;\;\;\;\;+\frac{1}{2}\left(f_{3}k_{3}x_{3}v_{1,3}\right)\left(f_{1,3}x_{1,3}+{f_{1,4}x}_{1,4}+{2f_{1,1}x}_{1,1}+c_{3,4}f_{3,4}x_{3,4}+\left(1-c_{1,2}\right){f_{1,2}x}_{1,2}\right)\\ \left(8\right)\;Tx_{1,4}^{\prime }=\frac{1}{2}\left({f_{1}k}_{1}x_{1}v_{1,4}\right)\left(f_{1,4}x_{1,4}+{f_{2,4}x}_{2,4}+{2f_{4,4}x}_{4,4}+c_{1,2}{f_{1,2}x}_{1,2}+\left(1-c_{3,4}\right){f_{3,4}x}_{3,4}\right)\\ \;\;\;\;\;\;\;\;\;\;\;\;\;\;\;\;\;+\frac{1}{2}\left(f_{4}k_{4}x_{4}v_{1,4}\right)\left(f_{1,3}x_{1,3}+{f_{1,4}x}_{1,4}+{2f_{1,1}x}_{1,1}+c_{3,4}f_{3,4}x_{3,4}+\left(1-c_{1,2}\right){f_{1,2}x}_{1,2}\right)\\ \begin{array}{l} \left(9\right)\;Tx_{2,2}^{\prime }=\frac{1}{2}\left({f_{2}k}_{2}x_{2}v_{2,2}\right)\left(f_{2,3}x_{2,3}+f_{2,4}x_{2,4}+{2f_{2,2}x}_{2,2}+c_{3,4}f_{3,4}x_{3,4}+\left(1-c_{1,2}\right)f_{1,2}x_{1,2}\right)\\ {\left(10\right)\;Tx}_{2,3}^{\prime }=\frac{1}{2}\left({f_{2}k}_{2}x_{2}v_{2,3}\right)\left(f_{1,3}x_{1,3}+{f_{2,3}x}_{2,3}+{2f_{3,3}x}_{3,3}+c_{1,2}{f_{1,2}x}_{1,2}+\left(1-c_{3,4}\right){f_{3,4}x}_{3,4}\right)\\ \;\;\;\;\;\;\;\;\;\;\;\;\;\;\;\;\;+\frac{1}{2}\left(f_{3}k_{3}x_{3}v_{2,3}\right)\left(f_{2,3}x_{2,3}+{f_{2,4}x}_{2,4}+{2f_{2,2}x}_{2,2}+c_{3,4}f_{3,4}x_{3,4}+\left(1-c_{1,2}\right){f_{1,2}x}_{1,2}\right)\\ \begin{array}{l} \left(11\right)\;Tx_{2,4}^{\prime }=\frac{1}{2}\left({f_{2}k}_{2}x_{2}v_{2,4}\right)\left(f_{1,4}x_{1,4}+{f_{2,4}x}_{2,4}+{2f_{4,4}x}_{4,4}+c_{1,2}{f_{1,2}x}_{1,2}+\left(1-c_{3,4}\right){f_{3,4}x}_{3,4}\right)\\ \;\;\;\;\;\;\;\;\;\;\;\;\;\;\;\;\;+\frac{1}{2}\left(f_{4}k_{4}x_{4}v_{2,4}\right)\left(f_{2,3}x_{2,3}+{f_{2,4}x}_{2,4}+{2f_{2,2}x}_{2,2}+c_{3,4}{f_{3,4}x}_{3,4}+\left(1-c_{1,2}\right){f_{1,2}x}_{1,2}\right)\\ \left(12\right)\;Tx_{3,3}^{\prime }=\frac{1}{2}\left({f_{3}k}_{3}x_{3}v_{3,3}\right)\left(f_{1,3}x_{1,3}+{f_{2,3}x}_{2,3}+{2f_{3,3}x}_{3,3}+c_{1,2}{f_{1,2}x}_{1,2}+\left(1-c_{3,4}\right)f_{3,4}x_{3,4}\right)\\ \begin{array}{l} \left(13\right)\;Tx_{3,4}^{\prime }=\frac{1}{2}\left({f_{3}k}_{3}x_{3}v_{3,4}\right)\left({f_{1,4}x}_{1,4}+{f_{2,4}x}_{2,4}+{2f_{4,4}x}_{4,4}+c_{1,2}{f_{1,2}x}_{1,2}+\left(1-c_{3,4}\right){f_{3,4}x}_{3,4}\right)\\ \;\;\;\;\;\;\;\;\;\;\;\;\;\;\;\;\;+\frac{1}{2}\left(f_{4}k_{4}x_{4}v_{3,4}\right)\left(f_{1,3}x_{1,3}+{f_{2,3}x}_{2,3}+{2f_{3,3}x}_{3,3}+c_{1,2}f_{1,2}x_{1,2}+\left(1-c_{3,4}\right){f_{3,4}x}_{3,4}\right)\\ \left(14\right)\;Tx_{4,4}^{\prime }=\frac{1}{2}\left(f_{4}k_{4}x_{4}v_{4,4}\right)\left(f_{1,4}x_{1,4}+{f_{2,4}x}_{2,4}+{2f_{4,4}x}_{4,4}+c_{1,2}{f_{1,2}x}_{1,2}+\left(1-c_{3,4}\right)f_{3,4}x_{3,4}\right) \end{array} \end{array} \end{array} \end{array} \end{array} \end{array} \end{array}$$

where T is the sum of the right side of the system of equations and the next generation’s genotypic frequencies are denoted with primes. Iteration of this single generation system allows graphical analysis of short-term evolution of *D. pseudoobscura Sex Ratio* system.

The behavior of this model was examined with experimentally determined parameters and several simplifying assumptions. First, relative viability and fertility for genotypes involving *X_ST_* and *X_SR_* were taken from direct measurements in Curtsinger and Feldman (1980) and are assumed to be recessive such that $v_{1,2}=f_{1,2}=1$. Second, relative viabilities and fertilities were assumed to be products of multiplicative fitness effects; such that when the 26-Mb *SR* haplotype was disrupted by recombination, then $v_{2,2}=v_{2,3}v_{2,4}$ and $f_{2,2}=f_{2,3}f_{2,4}$. Furthermore, relative viability and fertility of recombinant *X* chromosomes in the homozygous state was proportional to the fraction of the 26-Mb *SR* haplotype retained, such that $v_{2,3}=\sqrt[{3}]{{\left(v_{2,2}\right)}^{2}}$, $v_{2,4}=\sqrt[{3}]{\left(v_{2,2}\right)}$, $f_{2,3}=\sqrt[{3}]{{\left(f_{2,2}\right)}^{2}}$, and $f_{2,4}=\sqrt[{3}]{\left(f_{2,2}\right)}$. Following the same logic for hemizygote, relative viability and fertilities were $v_{2}=v_{3}v_{4}=\sqrt[{3}]{{\left(v_{2}\right)}^{2}}\;\sqrt[{3}]{\left(v_{2}\right)}$ and $f_{2}=f_{3}f_{4}=\sqrt[{3}]{{\left(f_{2}\right)}^{2}}\;\sqrt[{3}]{\left(f_{2}\right)}$. All full list numerical values for viability and fertility parameters are given in Table A1. Third, the *X*-linked segregation distortion (male genotype-specific proportion of female offspring sired) was $k_{1}=0.5$, $k_{2}=0.5$, $k_{3}=0.8$, and $k_{4}=1.0$ based on experimental estimates. Here, an averaged $k_{3}$ was used to account for polymorphic autosomal suppressors in natural populations that can alter the distortion strength of *X_BM_*. Finally, recombination rates were based on the experimental results reported in this study; however, 2 different models of recombination [reciprocal exchange due to meiotic crossing-over and unequal mitotic exchange in female germline stem cells (GSCs)] were investigated.

Meiotic crossing-over was modeled as reciprocal exchange using the experiment-wide rate of 0.00779 events per offspring for both directions of recombination $c_{1,2}$ (producing *X_BM_* and *X_TO_*) and $c_{3,4}$ (producing *X_ST_* and *X_SR_*). The “back” recombination rate $c_{3,4}$ (heterozygous *X_BM_*/*X_TO_* females generating *X_ST_* or *X_SR_* progeny) has not been experimentally estimated. However, the relevant female genotype $x_{3,4}$ only occurs on the order of 1 in 1,000,000 individuals in the natural population. Meiotic crossing-over produces system of Equation (A1) derived above.

Unequal mitotic exchange in the female GSCs was modeled by substituting the “forward” recombination rate ($c_{1,2}$ producing *X_BM_* and *X_TO_* from *X_ST_*/*X_SR_* female heterozygotes) with complementary class-specific rates $c_{3}$ (generating only *X_BM_*) and $c_{4}$ (generating only *X_TO_*) into the relevant columns of the mating table. Complementary class-specific rates ($c_{3}=0.00715$ and $c_{4}=0.0182$) were calculated from experimental recovery rates in the protected heterozygous state (i.e. from female progeny only). The “back” recombination rate $c_{3,4}$ was not altered as the relevant genotype $x_{3,4}$ was exceedingly rare. Finally, relative viability of hemizygous *X_BM_* and *X_TO_* was estimated by deficit of recombinant male versus female progeny observed in experimental data ($v_{3}=0.050$ and $v_{4}=0.392$). Therefore, the modified system of equations for unequal mitotic exchange was:

(A2) $$\begin{array}{l} \left(1\right)\;Tx_{1}^{\prime }=\frac{v_{1}}{2}\left({f_{1}x}_{1}\left({1-k}_{1}\right)+f_{2}x_{2}\left({1-k}_{2}\right)+f_{3}x_{3}\left({1-k}_{3}\right)+f_{4}x_{4}\left({1-k}_{4}\right)\right)\\ \;\;\;\;\;\;\;\;\;\;\;\;\;\;\;\;\;\times \left(f_{1,3}x_{1,3}+f_{1,4}x_{1,4}+{2f_{1,1}x}_{1,1}+{c_{3,4}f_{3,4}x}_{3,4}+{\left(1-c_{1,2}\right)f_{1,2}x}_{1,2}\right)\\ \left(2\right)\;Tx_{2}^{\prime }=\frac{v_{2}}{2}\left(f_{1}x_{1}\left({1-k}_{1}\right)+{f_{2}x}_{2}\left({1-k}_{2}\right)+{f_{3}x}_{3}\left({1-k}_{3}\right)+{f_{4}x}_{4}\left({1-k}_{4}\right)\right)\\ \;\;\;\;\;\;\;\;\;\;\;\;\;\;\;\;\;\times \left(f_{2,3}x_{2,3}+f_{2,4}x_{2,4}+{2f_{2,2}x}_{2,2}+{c_{3,4}f_{3,4}x}_{3,4}+{\left(1-c_{1,2}\right)f_{1,2}x}_{1,2}\right)\\ \begin{array}{l} \left(3\right)\;Tx_{3}^{\prime }=\frac{v_{3}}{2}\left(f_{1}x_{1}\left({1-k}_{1}\right)+f_{2}x_{2}\left({1-k}_{2}\right)+f_{3}x_{3}\left({1-k}_{3}\right)+f_{4}x_{4}\left({1-k}_{4}\right)\right)\\ \;\;\;\;\;\;\;\;\;\;\;\;\;\;\;\;\;\times \left(f_{1,3}x_{1,3}+f_{2,3}x_{2,3}+{2f_{3,3}x}_{3,3}+{c_{3}f_{1,2}x}_{1,2}+{\left(1-c_{3,4}\right)f_{3,4}x}_{3,4}\right)\\ \left(4\right)\;Tx_{4}^{\prime }=\frac{v_{4}}{2}\left(f_{1}x_{1}\left({1-k}_{1}\right)+f_{2}x_{2}\left({1-k}_{2}\right)+f_{3}x_{3}\left({1-k}_{3}\right)+f_{4}x_{4}\left({1-k}_{4}\right)\right)\\ \;\;\;\;\;\;\;\;\;\;\;\;\;\;\;\;\;\times \left(f_{1,4}x_{1,4}+f_{2,4}x_{2,4}+{2f_{4,4}x}_{4,4}+{c_{4}f_{1,2}x}_{1,2}+{\left(1-c_{3,4}\right)f_{3,4}x}_{3,4}\right)\\ \begin{array}{l} \left(5\right)\;Tx_{1,1}^{\prime }=\frac{1}{2}\left(f_{1}k_{1}x_{1}v_{1,1}\right)\left({f_{1,3}x}_{1,3}+{f_{1,4}x}_{1,4}+{2f_{1,1}x}_{1,1}+c_{3,4}{f_{3,4}x}_{3,4}+\left(1-c_{1,2}\right)f_{1,2}x_{1,2}\right)\\ \left(6\right)\;Tx_{1,2}^{\prime }=\frac{1}{2}\left({f_{1}k}_{1}x_{1}v_{1,2}\right)\left(f_{2,3}x_{2,3}+{f_{2,4}x}_{2,4}+{2f_{2,2}x}_{2,2}+c_{3,4}{f_{3,4}x}_{3,4}+\left(1-c_{1,2}\right)f_{1,2}x_{1,2}\right)\\ \;\;\;\;\;\;\;\;\;\;\;\;\;\;\;\;\;+\frac{1}{2}\left(f_{2}k_{2}x_{2}v_{1,2}\right)\left(f_{1,3}x_{1,3}+{f_{1,4}x}_{1,4}+{2f_{1,1}x}_{1,1}+c_{3,4}{f_{3,4}x}_{3,4}+\left(1-c_{1,2}\right)f_{1,2}x_{1,2}\right)\\ \begin{array}{l} \left(7\right)\;Tx_{1,3}^{\prime }=\frac{1}{2}\left({f_{1}k}_{1}x_{1}v_{1,3}\right)\left(f_{1,3}x_{1,3}+{f_{2,3}x}_{2,3}+{2f_{3,3}x}_{3,3}+c_{3}{f_{1,2}x}_{1,2}+\left(1-c_{3,4}\right){f_{3,4}x}_{3,4}\right)\\ \;\;\;\;\;\;\;\;\;\;\;\;\;\;\;\;\;+\frac{1}{2}\left(f_{3}k_{3}x_{3}v_{1,3}\right)\left(f_{1,3}x_{1,3}+{f_{1,4}x}_{1,4}+{2f_{1,1}x}_{1,1}+c_{3,4}f_{3,4}x_{3,4}+\left(1-c_{1,2}\right){f_{1,2}x}_{1,2}\right)\\ \left(8\right)\;Tx_{1,4}^{\prime }=\frac{1}{2}\left({f_{1}k}_{1}x_{1}v_{1,4}\right)\left(f_{1,4}x_{1,4}+{f_{2,4}x}_{2,4}+{2f_{4,4}x}_{4,4}+c_{4}{f_{1,2}x}_{1,2}+\left(1-c_{3,4}\right){f_{3,4}x}_{3,4}\right)\\ \;\;\;\;\;\;\;\;\;\;\;\;\;\;\;\;\;+\frac{1}{2}\left(f_{4}k_{4}x_{4}v_{1,4}\right)\left(f_{1,3}x_{1,3}+{f_{1,4}x}_{1,4}+{2f_{1,1}x}_{1,1}+c_{3,4}f_{3,4}x_{3,4}+\left(1-c_{1,2}\right){f_{1,2}x}_{1,2}\right)\\ \begin{array}{l} \left(9\right)\;Tx_{2,2}^{\prime }=\frac{1}{2}\left({f_{2}k}_{2}x_{2}v_{2,2}\right)\left(f_{2,3}x_{2,3}+f_{2,4}x_{2,4}+{2f_{2,2}x}_{2,2}+c_{3,4}f_{3,4}x_{3,4}+\left(1-c_{1,2}\right)f_{1,2}x_{1,2}\right)\\ \left(10\right)\;{Tx}_{2,3}^{\prime }=\frac{1}{2}\left({f_{2}k}_{2}x_{2}v_{2,3}\right)\left(f_{1,3}x_{1,3}+{f_{2,3}x}_{2,3}+{2f_{3,3}x}_{3,3}+c_{3}{f_{1,2}x}_{1,2}+\left(1-c_{3,4}\right){f_{3,4}x}_{3,4}\right)\\ \;\;\;\;\;\;\;\;\;\;\;\;\;\;\;\;\;+\frac{1}{2}\left(f_{3}k_{3}x_{3}v_{2,3}\right)\left(f_{2,3}x_{2,3}+{f_{2,4}x}_{2,4}+{2f_{2,2}x}_{2,2}+c_{3,4}f_{3,4}x_{3,4}+\left(1-c_{1,2}\right){f_{1,2}x}_{1,2}\right)\\ \begin{array}{l} \left(11\right)\;Tx_{2,4}^{\prime }=\frac{1}{2}\left({f_{2}k}_{2}x_{2}v_{2,4}\right)\left(f_{1,4}x_{1,4}+{f_{2,4}x}_{2,4}+{2f_{4,4}x}_{4,4}+c_{4}{f_{1,2}x}_{1,2}+\left(1-c_{3,4}\right){f_{3,4}x}_{3,4}\right)\\ \;\;\;\;\;\;\;\;\;\;\;\;\;\;\;\;\;+\frac{1}{2}\left(f_{4}k_{4}x_{4}v_{2,4}\right)\left(f_{2,3}x_{2,3}+{f_{2,4}x}_{2,4}+{2f_{2,2}x}_{2,2}+c_{3,4}{f_{3,4}x}_{3,4}+\left(1-c_{1,2}\right){f_{1,2}x}_{1,2}\right)\\ \begin{array}{l} \left(12\right)\;Tx_{3,3}^{\prime }=\frac{1}{2}\left({f_{3}k}_{3}x_{3}v_{3,3}\right)\left(f_{1,3}x_{1,3}+{f_{2,3}x}_{2,3}+{2f_{3,3}x}_{3,3}+c_{3}{f_{1,2}x}_{1,2}+\left(1-c_{3,4}\right)f_{3,4}x_{3,4}\right)\\ \begin{array}{l} \left(13\right)\;Tx_{3,4}^{\prime }=\frac{1}{2}\left({f_{3}k}_{3}x_{3}v_{3,4}\right)\left({f_{1,4}x}_{1,4}+{f_{2,4}x}_{2,4}+{2f_{4,4}x}_{4,4}+c_{4}{f_{1,2}x}_{1,2}+\left(1-c_{3,4}\right){f_{3,4}x}_{3,4}\right)\\ \;\;\;\;\;\;\;\;\;\;\;\;\;\;\;\;\;+\frac{1}{2}\left(f_{4}k_{4}x_{4}v_{3,4}\right)\left(f_{1,3}x_{1,3}+{f_{2,3}x}_{2,3}+{2f_{3,3}x}_{3,3}+c_{3}f_{1,2}x_{1,2}+\left(1-c_{3,4}\right){f_{3,4}x}_{3,4}\right)\\ \left(14\right)\;Tx_{4,4}^{\prime }=\frac{1}{2}\left(f_{4}k_{4}x_{4}v_{4,4}\right)\left(f_{1,4}x_{1,4}+{f_{2,4}x}_{2,4}+{2f_{4,4}x}_{4,4}+c_{4}{f_{1,2}x}_{1,2}+\left(1-c_{3,4}\right)f_{3,4}x_{3,4}\right) \end{array} \end{array} \end{array} \end{array} \end{array} \end{array} \end{array} \end{array}$$

where, as before, T is the sum of the right side of equations, primes denote next generation’s genotypic frequencies, and the altered terms $\left(c_{3},\;c_{4},\;v_{3},\;\mathrm{and}\;v_{4}\right)$ are bolded for convenience.

Thorough equilibrium and stability analysis of the full 14 variable population genetic model is beyond the scope of this article. Instead, equilibrium frequencies were solved for by iteration of the system of recursion equations. Initial genotypic frequencies were taken from Beckenbach’s (1996) direct cytological observation of *SR* inversion complement for 694 *X* chromosomes sampled from Tucson and Bear Creek, AZ, USA. The equilibrium frequencies are reported in Table 6. This procedure was then repeated with a range of initial allele frequencies (0.01–0.99) for *X_SR_*, *X_BM_*, and *X_TO_* under both alternative scenarios: meiotic crossing-over in oocyte or unequal mitotic exchange in GSCs. In all cases, the genotypic frequencies converged on the same values in less than 1,000 generations (Fig. 5).

**Table A1.** Viability and fertility parameter values for the recombination extension of Edwards (1961) model.

	Viability			Fertility		
Genotype	Parameter	Estimate	Method	Parameter	Estimate	Method
*X_ST_Y*	*v_1_*	1.02	Empirical	*f_1_*	1.00	Empirical
*X_SR_Y*	*v_2_*	0.71	Empirical	*f_2_*	1.03	Empirical
*X_BM_Y*	*v_3_*	0.80	Calculated	*f_3_*	1.02	Calculated
*X_TO_Y*	*v_4_*	0.89	Calculated	*f_4_*	1.01	Calculated
*X_ST_X_ST_*	*v_1,1_*	1.24	Empirical	*f_1,1_*	0.87	Empirical
*X_ST_X_SR_*	*v_1,2_*	1.00	Defined	*f_1,2_*	1.00	Defined
*X_ST_X_BM_*	*v_1,3_*	1.07	Calculated	*f_1,3_*	0.95	Calculated
*X_ST_X_TO_*	*v_1,4_*	1.15	Calculated	*f_1,4_*	0.91	Calculated
*X_SR_X_SR_*	*v_2,2_*	0.55	Empirical	*f_2,2_*	0.68	Empirical
*X_SR_X_BM_*	*v_2,3_*	0.67	Calculated	*f_2,3_*	0.77	Calculated
*X_SR_X_TO_*	*v_2,4_*	0.82	Calculated	*f_2,4_*	0.88	Calculated
*X_BM_X_BM_*	*v_3,3_*	0.72	Calculated	*f_3,3_*	0.73	Calculated
*X_BM_X_TO_*	*v_3,4_*	1.00	Calculated	*f_3,4_*	1.00	Calculated
*X_TO_X_TO_*	*v_4,4_*	0.95	Calculated	*f_4,4_*	0.80	Calculated

Parameter is classified as “defined” if its value is set by the modeling framework, “empirical” if directly measured in Curtsinger and Feldman (1980), and “calculated” if based on the proportional division of Curtsinger and Feldman (1980) values.
