# Supplementary material for: Mitotic exchange in female germline stem cells is the major source of Sex Ratio chromosome recombination in Drosophila pseudoobscura
Source: G3 (Bethesda). 2022 Oct 4;12(12):jkac264. doi: 10.1093/g3journal/jkac264 (PMC9713450; doi:10.1093/g3journal/jkac264)
Supplement: jkac264_Supplementary_File_S2 [file jkac264_supplementary_file_s2.pdf]

File S2. Supplemental tables for Mitotic exchange in female germline stem cells is the major source of *Sex Ratio* chromosome recombination in *Drosophila pseudoobscura*

Table S1. Experimental genotypes Page 2

Table S2. ANOVA tables Page 3

Table S3. Poisson goodness-of-fit tables Page 4

Table S4. Binomial goodness-of-fit tables Page 5

Table S5. χ^2 tests for complementary recombinants Page 6

Table S1. Experimental genotypes in this study. Experimental F_1 female heterozygotes (X_{SR}/X_{ST}) were produced as all possible combinations 3 X_{SR} isolate, 3 X_{ST} marker combinations, and 3 genetic background $(3 \times 3 \times 3)$. All genotypes that would produce inbred genetic backgrounds were excluded $(3 \times 3 \times 3 - 9)$, generating a total of 18 unique outbred experimental genotypes for testing recombination rates.

Heterozygous X Chromosome Genotype	Heterozygous Autosomal Genotype
SR Isolate KBNP2 / y^1, se^1, sh^1	14011-0121.06 / 14011-0121.08
SR Isolate KBNP2 / y^1, se^1, sh^1	14011-0121.06 / Phadnis Lab Line 2020
SR Isolate KBNP2 / se^1, ll^1, sp^1, tt^1	14011-0121.06 / 14011-0121.08
SR Isolate KBNP2 / se^1, ll^1, sp^1, tt^1	14011-0121.08 / Phadnis Lab Line 2020
SR Isolate KBNP2 / se^1, sh^1	14011-0121.06 / Phadnis Lab Line 2020
SR Isolate KBNP2 / se^1, sh^1	14011-0121.08 / Phadnis Lab Line 2020
SR Isolate Z6 / y^1, se^1, sh^1	14011-0121.06 / 14011-0121.08
SR Isolate Z6 / y^1, se^1, sh^1	14011-0121.06 / Phadnis Lab Line 2020
SR Isolate Z6 / se^1, ll^1, sp^1, tt^1	14011-0121.06 / 14011-0121.08
SR Isolate Z6 / se^1, ll^1, sp^1, tt^1	14011-0121.08 / Phadnis Lab Line 2020
SR Isolate Z6 / se^1, sh^1	14011-0121.06 / Phadnis Lab Line 2020
SR Isolate Z6 / se^1, sh^1	14011-0121.08 / Phadnis Lab Line 2020
SR Isolate Z8 / y^1, se^1, sh^1	14011-0121.06 / 14011-0121.08
SR Isolate Z8 / y^1, se^1, sh^1	14011-0121.06 / Phadnis Lab Line 2020
SR Isolate Z8 / se^1, ll^1, sp^1, tt^1	14011-0121.06 / 14011-0121.08
SR Isolate Z8 / se^1, ll^1, sp^1, tt^1	14011-0121.08 / Phadnis Lab Line 2020
SR Isolate Z8 / se^1, sh^1	14011-0121.06 / Phadnis Lab Line 2020
SR Isolate Z8 / se^1, sh^1	14011-0121.08 / Phadnis Lab Line 2020

Table S2. Sex-stratified analysis of the effect of X_{SR} isolate and genetic background on recombination rate. A) ANOVA table with recombination rates determined by female F_2 progeny only. No statistically significant effects were detected in this analysis. B) ANOVA table with recombination rates determined by male F_2 progeny only. No statistically significant effects were detected in this analysis.

A)

Source of Variation	*df*	*SS*	*MS*	F_s	*p-value*
X_{SR} Chromosome Isolate	2	26.12	13.06	0.46	0.63
Genetic Background	2	61.09	30.55	1.09	0.34
Residuals	115	3236.78	28.15		
Total	119	3323.99			

B)

Source of Variation	*df*	*SS*	*MS*	F_s	*p-value*
X_{SR} Chromosome Isolate	2	9.45	4.73	0.42	0.66
Genetic Background	2	22.18	11.09	0.98	0.38
Residuals	115	1307.57	11.37		
Total	119	1339.20			

Table S3. Sex-stratified analysis of clustering among F_1 females. A) A statistically significant χ^2 goodness-of-fit test for number of female F_2 recombinants per single-female cross $\left(\chi^2_{[3]} = 8.32, p = 0.0179\right)$. Expectations generated from the Poisson distribution with $\lambda = 0.01136$. B) A statistically significant χ^2 goodness-of-fit test for number of male F_2 recombinants per single-female cross $\left(\chi^2_{[2]} = 5.57, p = 0.0308\right)$. Expectations generated from the Poisson distribution with $\lambda = 0.003780$. In sex-stratified analysis, too few recombinants were recovered to support five classes in the χ^2 goodness-of-fit tests, therefore all single-female crosses producing greater than or equal to three female recombinants and greater than or equal to two male recombinants were pooled, this was a conservative procedure.

A)

Category	*Expected*	*Observed*	$(Obs\text{-}Exp)^2/Exp$
0 Recombinants	66.95	77	1.51
1 Recombinant	38.52	29	2.35
2 Recombinants	11.64	8	1.14
≥3 Recombinants	2.90	6	3.33

B)

Category	*Expected*	*Observed*	$(Obs\text{-}Exp)^2/Exp$
0 Recombinants	100.84	105	0.17
1 Recombinant	17.44	11	2.38
≥2 Recombinants	1.72	4	3.02

Table S4. Sex-stratified analysis of clustering within F_1 females producing multiple recombinants. B) A statistically significant χ^2 goodness-of-fit test for the proportion (q) of female F_2 recombinants from a single-female cross that carried X_{TO} $\left(\chi^2_{[4]} = 38.14, p = 7.96 \times 10^{-8}\right)$. C) A statistically significant χ^2 goodness-of-fit test for the proportion of female F_2 recombinants from a single-female cross that carried X_{TO} $\left(\chi^2_{[2]} = 15.69, p = 1.95 \times 10^{-4}\right)$. Only four single-female crosses produced multiple male recombinants so only three bins were used in the χ^2 goodness-of-fit test.

A)

Category	*Expected*	*Observed*	*(Obs-Exp)^2/Exp*
$0.0 = q$	2.47	1	2.47
$0.0 < q \leq 0.3$	1.67	1	0.27
$0.3 < q \leq 0.6$	5.71	0	5.71
$0.6 < q \leq 0.9$	1.67	1	0.27
$0.9 < q \leq 1.0$	2.47	11	29.41

B)

Category	*Expected*	*Observed*	*(Obs-Exp)^2/Exp*
$0.0 = q$	0.81	0	0.81
$0.0 < q < 1.0$	2.38	0	2.38
$1.0 = q$	0.81	4	12.50

Table S5. Observed recovery rates of complementary recombinants were not independent of F_2 progeny sex even after estimating different rates for complementary classes from the data. The observed data differed from uniform discrete expectations with statistical significance $\left(\chi^2_{[3]} = 55.9, p = 2.15 \times 10^{-12}\right)$ (Main text Table 5). A) The observed data still differed from expectations when using one more degree of freedom to estimate different rates of production from the data for complementary recombinant classes $\left(\chi^2_{[2]} = 34.7, p = 1.06 \times 10^{-7}\right)$. B) Observed recovery rates of recombinants did not meet expectations even after estimating different rates for complementary classes and sexes from the data $\left(\chi^2_{[1]} = 5.04, p = 0.0143\right)$ using two more degree of freedom for additional parameters drawn from data.

A)

Category	*Expected*	*Observed*	*(Obs-Exp)^2/ Exp*
X_{BM}, Female	10.5	20	8.60
X_{BM}, Male	10.5	1	8.60
X_{TO}, Female	35.5	51	6.77
X_{TO}, Male	35.5	20	6.77
			χ2 = 30.73

B)

Category	*Expected*	*Observed*	*(Obs-Exp)^2/ Exp*
X_{BM}, Female	16.2	20	0.89
X_{BM}, Male	4.8	1	3.00
X_{TO}, Female	54.8	51	0.26
X_{TO}, Male	16.2	20	0.89
			χ2 = 5.04
