# Supplementary material for: Mitotic exchange in female germline stem cells is the major source of Sex Ratio chromosome recombination in Drosophila pseudoobscura
Source: G3 (Bethesda). 2022 Oct 4;12(12):jkac264. doi: 10.1093/g3journal/jkac264 (PMC9713450; doi:10.1093/g3journal/jkac264)
Supplement: jkac264_Supplementary_File_S3 [file jkac264_supplementary_file_s3.pdf]

File S3. Experimental design matrix with illustration of recombination experiment crossing scheme. Following slides are color-coded to match 3 x 3 x 3 - 9 factorial design to generate each of 18 unique F_1 experimental genotypes.

	Sex Ratio Chromosome Isolate	*KBPN2*	*KBPN2*	*KBPN2*	*Z8*	*Z8*	*Z8*	*Z6*	*Z6*	*Z6*
	Paternal Genetic Background	GB_1	GB_2	GB_3	GB_1	GB_2	GB_3	GB_1	GB_2	GB_3
Maternal Genotype	y^1 se^1 sh^1; 14011-0121.06 (GB_1)	×	1	2	×	3	4	×	5	6
	se^1 ll^1 sp^1 tt^1; 14011-0121.08 (GB_2)	7	×	8	9	×	10	11	×	12
	se^1 sh^1; Lab Line 2020 (GB_3)	13	14	×	15	16	×	17	18	×

× Indicates heterozygous genotype with inbred genetic background not investigated

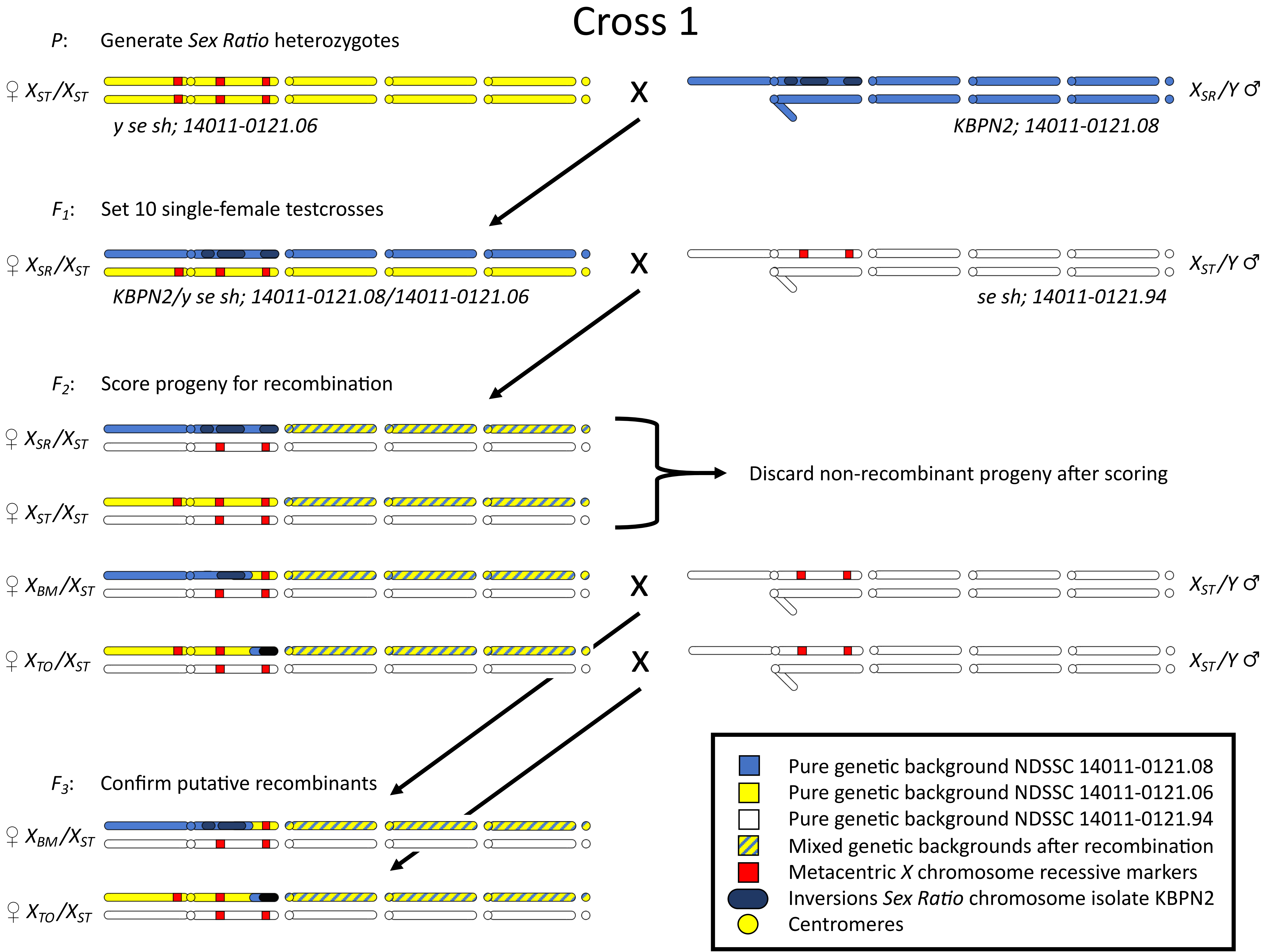
Cross 1
P: Generate Sex Ratio heterozygotes
♀ X_{ST}/X_{ST}
X
X_{SR}/Y ♂
y se sh; 14011-0121.06
KBPN2; 14011-0121.08
F1: Set 10 single-female testcrosses
♀ X_{SR}/X_{ST}
X
X_{ST}/Y ♂
KBPN2/y se sh; 14011-0121.08/14011-0121.06
se sh; 14011-0121.94
F2: Score progeny for recombination
♀ X_{SR}/X_{ST}
♀ X_{ST}/X_{ST}
Discard non-recombinant progeny after scoring
♀ X_{BM}/X_{ST}
X
X_{ST}/Y ♂
♀ X_{TO}/X_{ST}
X
X_{ST}/Y ♂
F3: Confirm putative recombinants
♀ X_{BM}/X_{ST}
♀ X_{TO}/X_{ST}
Pure genetic background NDSSC 14011-0121.08
Pure genetic background NDSSC 14011-0121.06
Pure genetic background NDSSC 14011-0121.94
Mixed genetic backgrounds after recombination
Metacentric X chromosome recessive markers
Inversions Sex Ratio chromosome isolate KBPN2
Centromeres

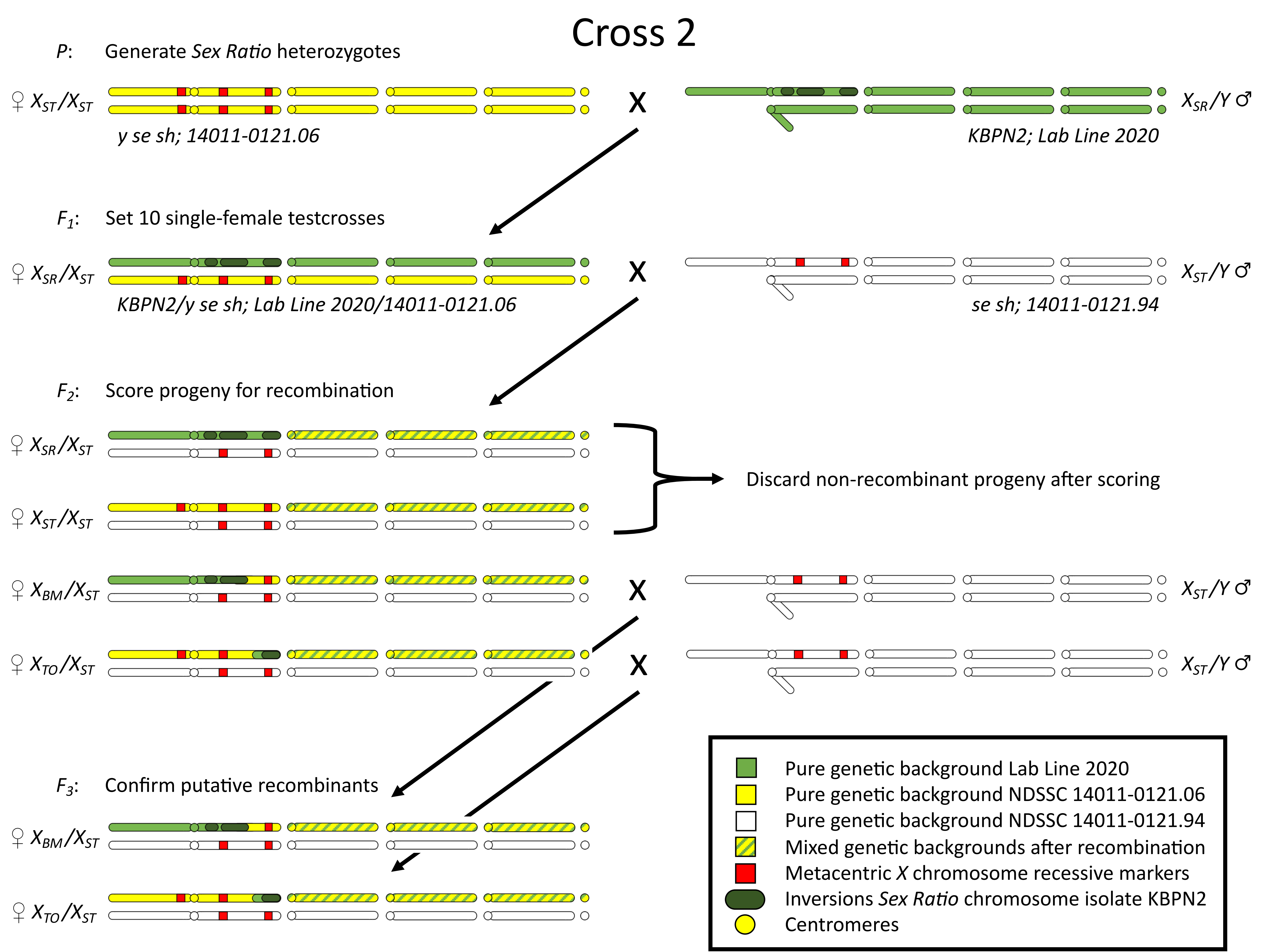
Cross 2
P: Generate Sex Ratio heterozygotes
♀ XST/XST
y se sh; 14011-0121.06
X
XSR/Y ♂
KBPN2; Lab Line 2020
F1: Set 10 single-female testcrosses
♀ XSR/XST
KBPN2/y se sh; Lab Line 2020/14011-0121.06
X
XST/Y ♂
se sh; 14011-0121.94
F2: Score progeny for recombination
♀ XSR/XST
♀ XST/XST
Discard non-recombinant progeny after scoring
♀ XBM/XST
X
XST/Y ♂
♀ XTO/XST
X
XST/Y ♂
F3: Confirm putative recombinants
♀ XBM/XST
♀ XTO/XST
Pure genetic background Lab Line 2020
Pure genetic background NDSSC 14011-0121.06
Pure genetic background NDSSC 14011-0121.94
Mixed genetic backgrounds after recombination
Metacentric X chromosome recessive markers
Inversions Sex Ratio chromosome isolate KBPN2
Centromeres

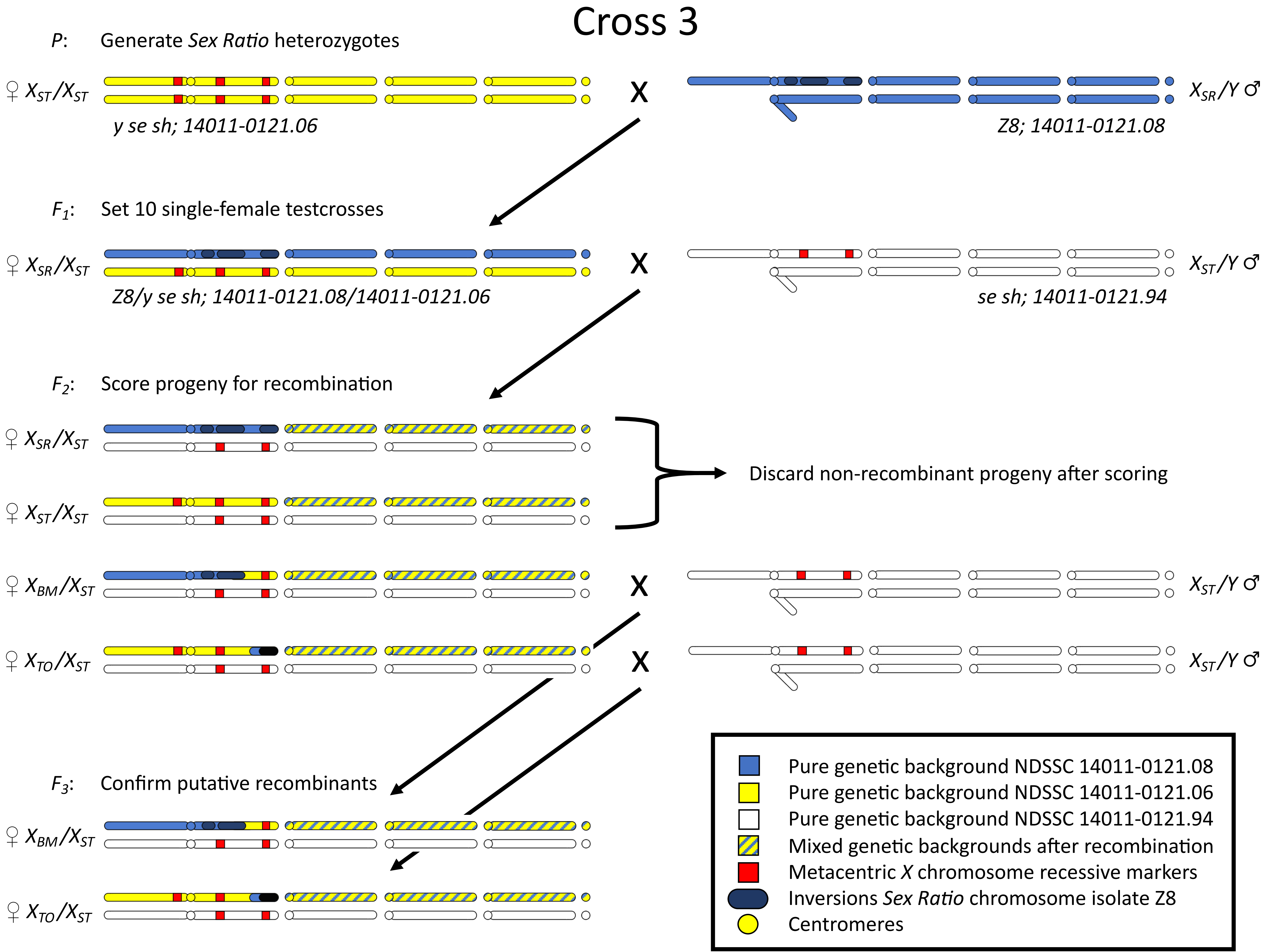
Cross 3
P: Generate Sex Ratio heterozygotes
♀ X_ST/X_ST
X
X_SR/Y ♂
y se sh; 14011-0121.06
Z8; 14011-0121.08
F1: Set 10 single-female testcrosses
♀ X_SR/X_ST
X
X_ST/Y ♂
Z8/y se sh; 14011-0121.08/14011-0121.06
se sh; 14011-0121.94
F2: Score progeny for recombination
♀ X_SR/X_ST
♀ X_ST/X_ST
Discard non-recombinant progeny after scoring
♀ X_BM/X_ST
X
X_ST/Y ♂
♀ X_TO/X_ST
X
X_ST/Y ♂
F3: Confirm putative recombinants
♀ X_BM/X_ST
♀ X_TO/X_ST
Pure genetic background NDSSC 14011-0121.08
Pure genetic background NDSSC 14011-0121.06
Pure genetic background NDSSC 14011-0121.94
Mixed genetic backgrounds after recombination
Metacentric X chromosome recessive markers
Inversions Sex Ratio chromosome isolate Z8
Centromeres

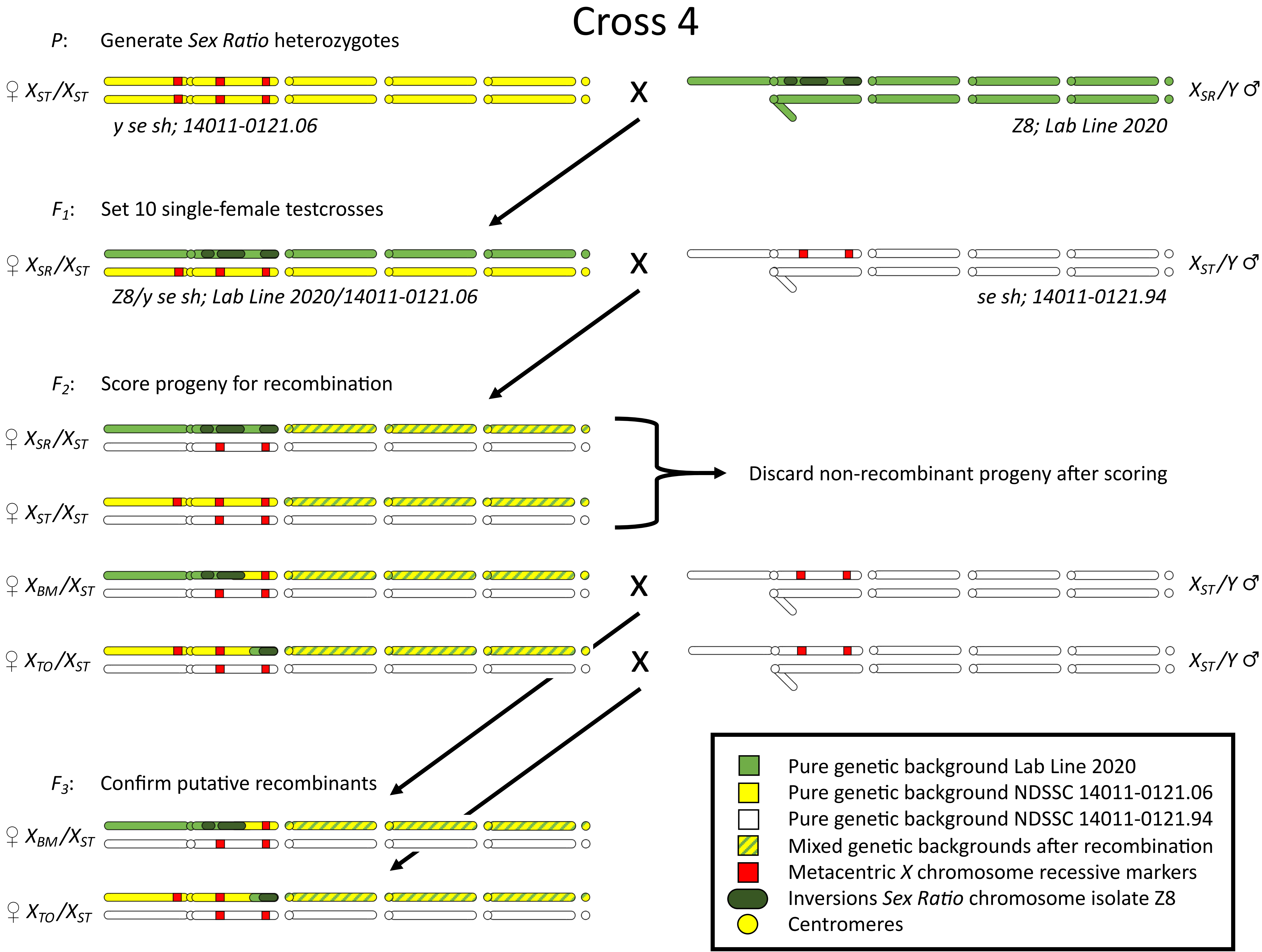

Cross 4
P: Generate Sex Ratio heterozygotes
♀ X_ST/X_ST
y se sh; 14011-0121.06
X
X_SR/Y ♂
Z8; Lab Line 2020
F1: Set 10 single-female testcrosses
♀ X_SR/X_ST
Z8/y se sh; Lab Line 2020/14011-0121.06
X
X_ST/Y ♂
se sh; 14011-0121.94
F2: Score progeny for recombination
♀ X_SR/X_ST
♀ X_ST/X_ST
Discard non-recombinant progeny after scoring
♀ X_BM/X_ST
X
X_ST/Y ♂
♀ X_TO/X_ST
X
X_ST/Y ♂
F3: Confirm putative recombinants
♀ X_BM/X_ST
♀ X_TO/X_ST
Pure genetic background Lab Line 2020
Pure genetic background NDSSC 14011-0121.06
Pure genetic background NDSSC 14011-0121.94
Mixed genetic backgrounds after recombination
Metacentric X chromosome recessive markers
Inversions Sex Ratio chromosome isolate Z8
Centromeres

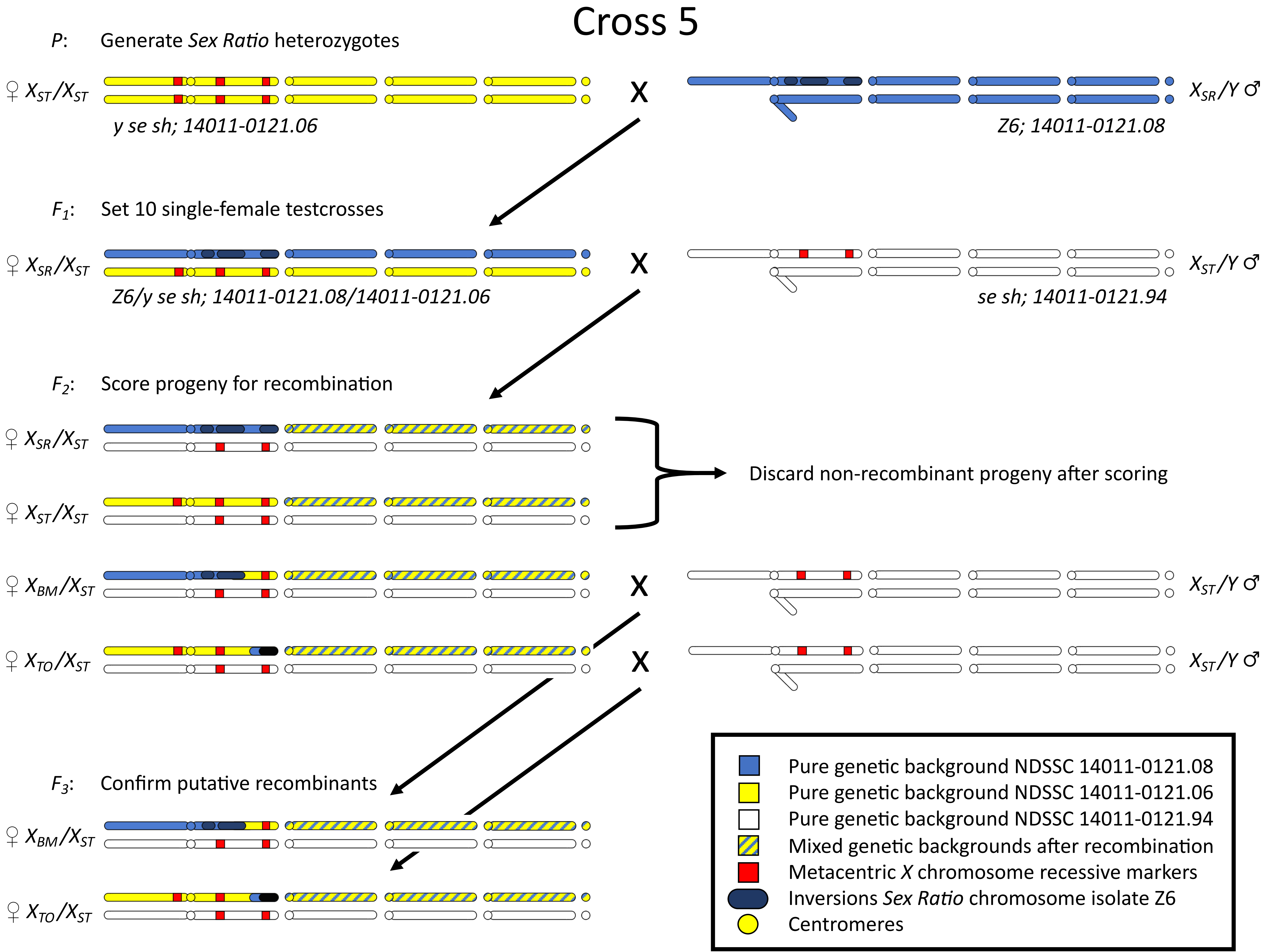
Cross 5
P: Generate Sex Ratio heterozygotes
♀ X_ST/X_ST
y se sh; 14011-0121.06
X
X_SR/Y ♂
Z6; 14011-0121.08
F1: Set 10 single-female testcrosses
♀ X_SR/X_ST
Z6/y se sh; 14011-0121.08/14011-0121.06
X
X_ST/Y ♂
se sh; 14011-0121.94
F2: Score progeny for recombination
♀ X_SR/X_ST
♀ X_ST/X_ST
Discard non-recombinant progeny after scoring
♀ X_BM/X_ST
X
X_ST/Y ♂
♀ X_TO/X_ST
X
X_ST/Y ♂
F3: Confirm putative recombinants
♀ X_BM/X_ST
♀ X_TO/X_ST
Pure genetic background NDSSC 14011-0121.08
Pure genetic background NDSSC 14011-0121.06
Pure genetic background NDSSC 14011-0121.94
Mixed genetic backgrounds after recombination
Metacentric X chromosome recessive markers
Inversions Sex Ratio chromosome isolate Z6
Centromeres

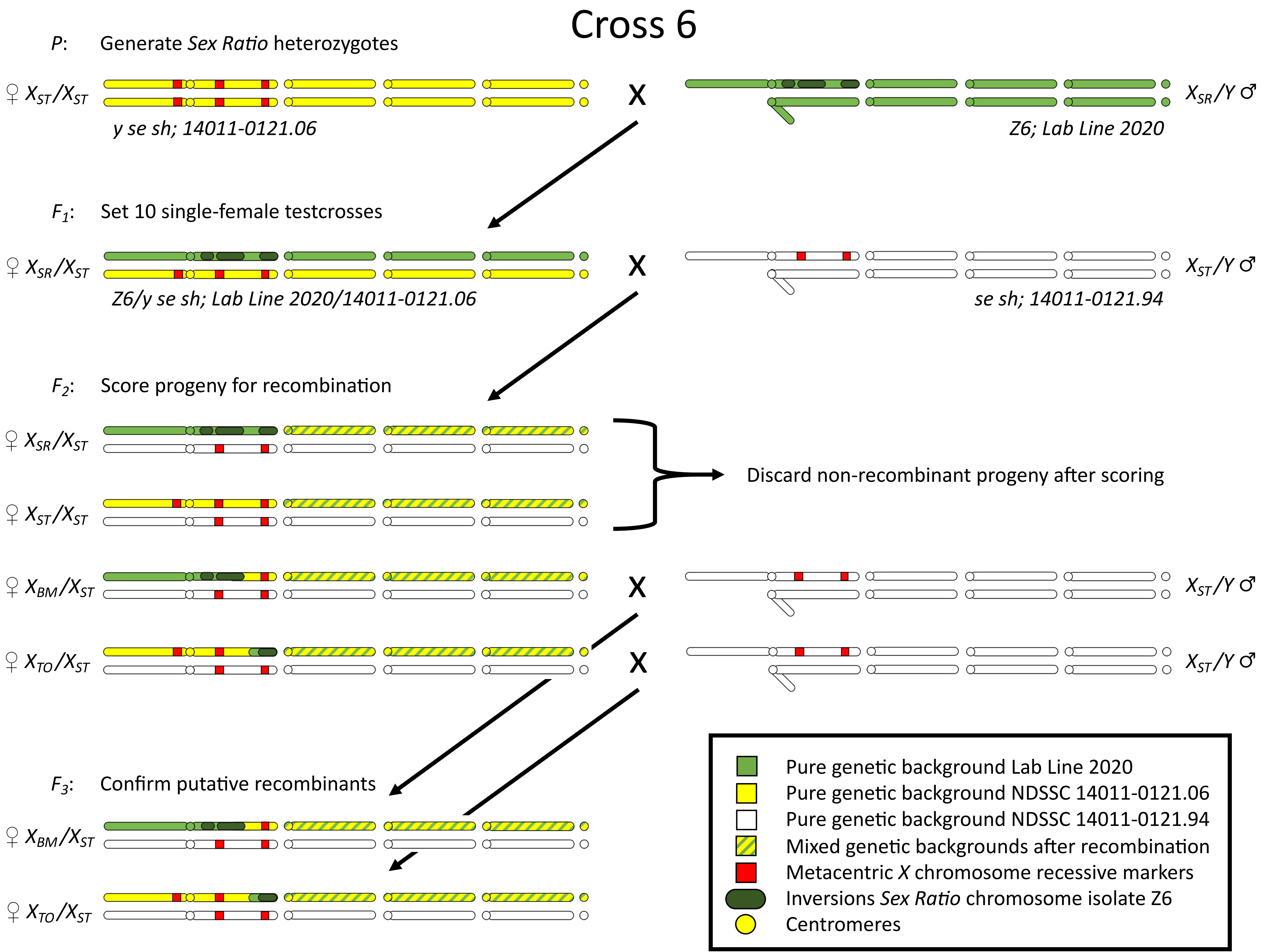

Cross 6
P: Generate Sex Ratio heterozygotes
♀ X_ST/X_ST
y se sh; 14011-0121.06
X
X_SR/Y ♂
Z6; Lab Line 2020
F_1: Set 10 single-female testcrosses
♀ X_SR/X_ST
Z6/y se sh; Lab Line 2020/14011-0121.06
X
X_ST/Y ♂
se sh; 14011-0121.94
F_2: Score progeny for recombination
♀ X_SR/X_ST
♀ X_ST/X_ST
Discard non-recombinant progeny after scoring
♀ X_BM/X_ST
X
X_ST/Y ♂
♀ X_TO/X_ST
X
X_ST/Y ♂
F_3: Confirm putative recombinants
♀ X_BM/X_ST
♀ X_TO/X_ST
Pure genetic background Lab Line 2020
Pure genetic background NDSSC 14011-0121.06
Pure genetic background NDSSC 14011-0121.94
Mixed genetic backgrounds after recombination
Metacentric X chromosome recessive markers
Inversions Sex Ratio chromosome isolate Z6
Centromeres

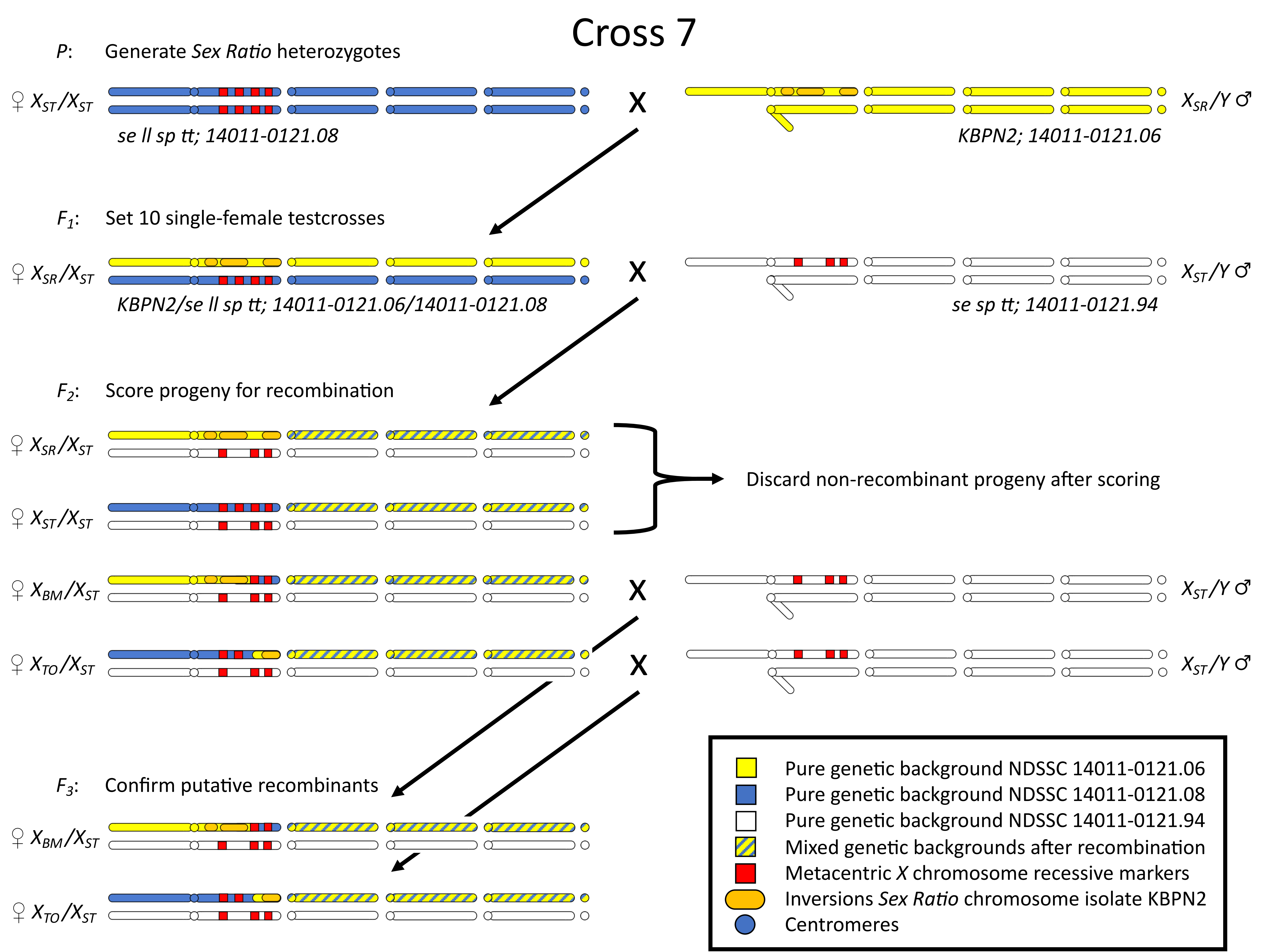
Cross 7
P: Generate Sex Ratio heterozygotes
♀ X_ST/X_ST
se ll sp tt; 14011-0121.08
X
X_SR/Y ♂
KBPN2; 14011-0121.06
F1: Set 10 single-female testcrosses
♀ X_SR/X_ST
KBPN2/se ll sp tt; 14011-0121.06/14011-0121.08
X
X_ST/Y ♂
se sp tt; 14011-0121.94
F2: Score progeny for recombination
♀ X_SR/X_ST
♀ X_ST/X_ST
Discard non-recombinant progeny after scoring
♀ X_BM/X_ST
X
X_ST/Y ♂
♀ X_TO/X_ST
X
X_ST/Y ♂
F3: Confirm putative recombinants
♀ X_BM/X_ST
♀ X_TO/X_ST
Pure genetic background NDSSC 14011-0121.06
Pure genetic background NDSSC 14011-0121.08
Pure genetic background NDSSC 14011-0121.94
Mixed genetic backgrounds after recombination
Metacentric X chromosome recessive markers
Inversions Sex Ratio chromosome isolate KBPN2
Centromeres

Cross 8

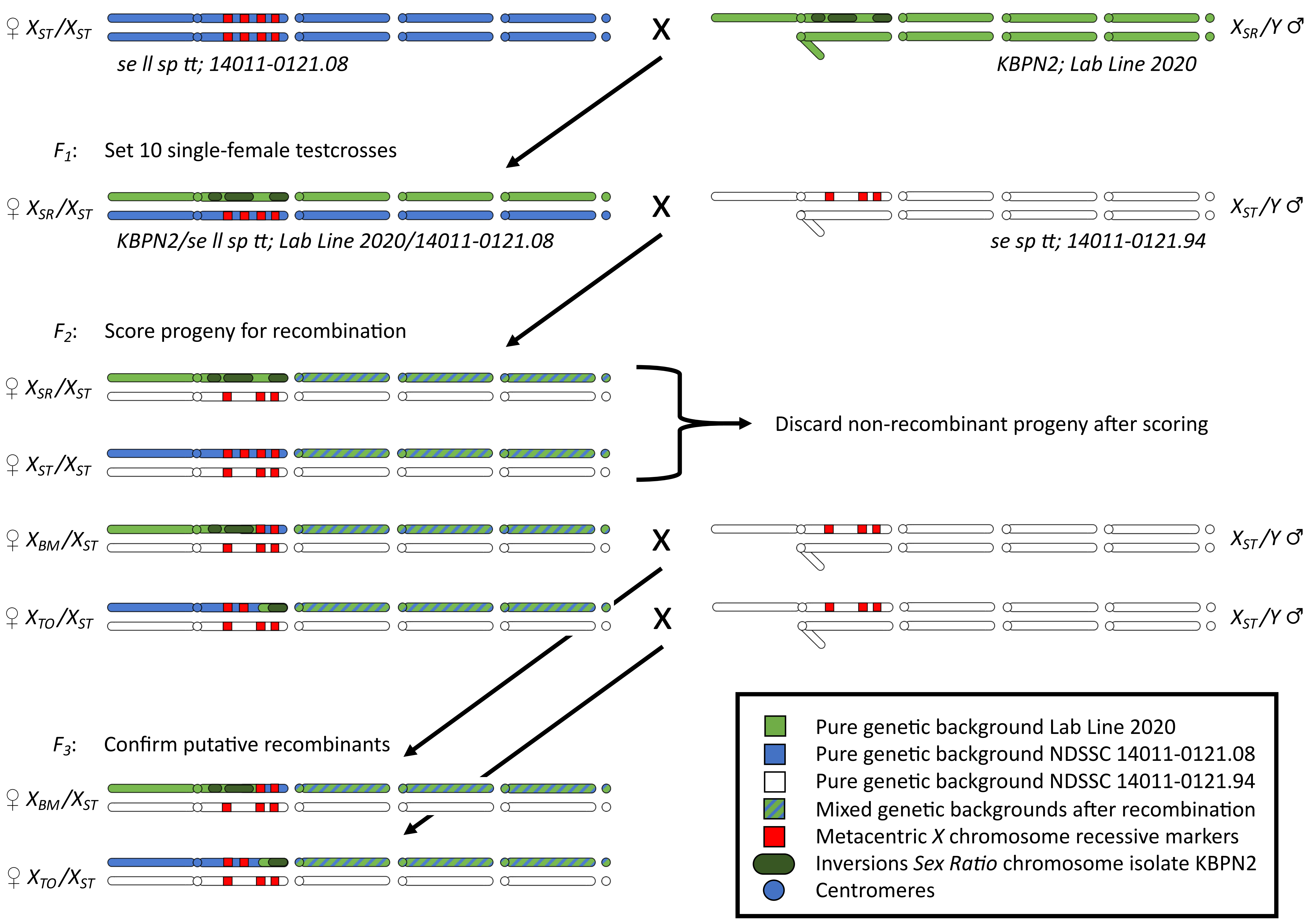

P: Generate Sex Ratio heterozygotes
♀ X_ST/X_ST
se ll sp tt; 14011-0121.08
X
X_SR/Y ♂
KBPN2; Lab Line 2020
F1: Set 10 single-female testcrosses
♀ X_SR/X_ST
KBPN2/se ll sp tt; Lab Line 2020/14011-0121.08
X
X_ST/Y ♂
se sp tt; 14011-0121.94
F2: Score progeny for recombination
♀ X_SR/X_ST
♀ X_ST/X_ST
Discard non-recombinant progeny after scoring
♀ X_BM/X_ST
X
X_ST/Y ♂
♀ X_TO/X_ST
X
X_ST/Y ♂
F3: Confirm putative recombinants
♀ X_BM/X_ST
♀ X_TO/X_ST
Pure genetic background Lab Line 2020
Pure genetic background NDSSC 14011-0121.08
Pure genetic background NDSSC 14011-0121.94
Mixed genetic backgrounds after recombination
Metacentric X chromosome recessive markers
Inversions Sex Ratio chromosome isolate KBPN2
Centromeres

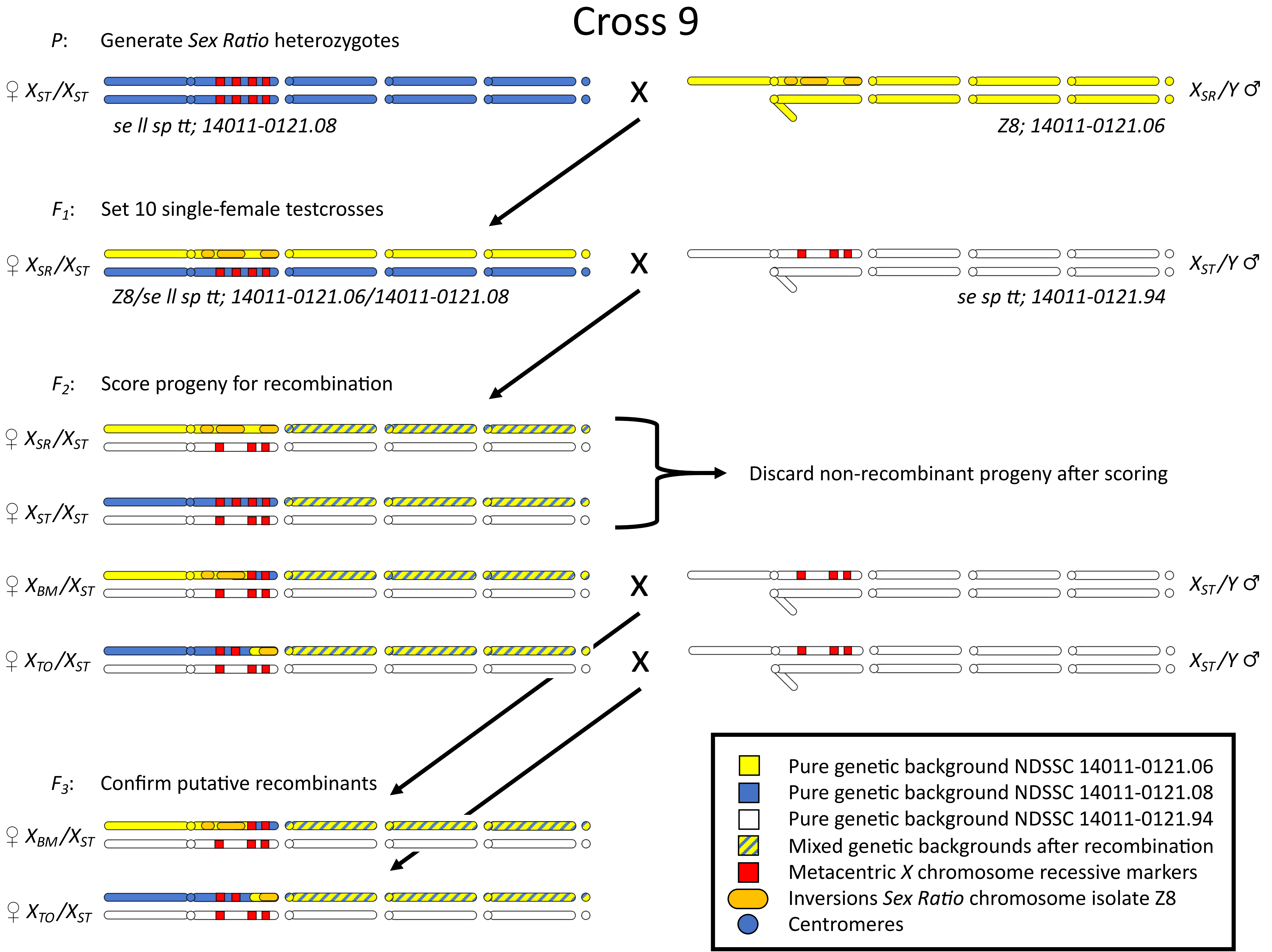
Cross 9
P: Generate Sex Ratio heterozygotes
♀ X_ST/X_ST
se ll sp tt; 14011-0121.08
X
X_SR/Y ♂
Z8; 14011-0121.06
F1: Set 10 single-female testcrosses
♀ X_SR/X_ST
Z8/se ll sp tt; 14011-0121.06/14011-0121.08
X
X_ST/Y ♂
se sp tt; 14011-0121.94
F2: Score progeny for recombination
♀ X_SR/X_ST
♀ X_ST/X_ST
Discard non-recombinant progeny after scoring
♀ X_BM/X_ST
X
X_ST/Y ♂
♀ X_TO/X_ST
X
X_ST/Y ♂
F3: Confirm putative recombinants
♀ X_BM/X_ST
♀ X_TO/X_ST
Pure genetic background NDSSC 14011-0121.06
Pure genetic background NDSSC 14011-0121.08
Pure genetic background NDSSC 14011-0121.94
Mixed genetic backgrounds after recombination
Metacentric X chromosome recessive markers
Inversions Sex Ratio chromosome isolate Z8
Centromeres

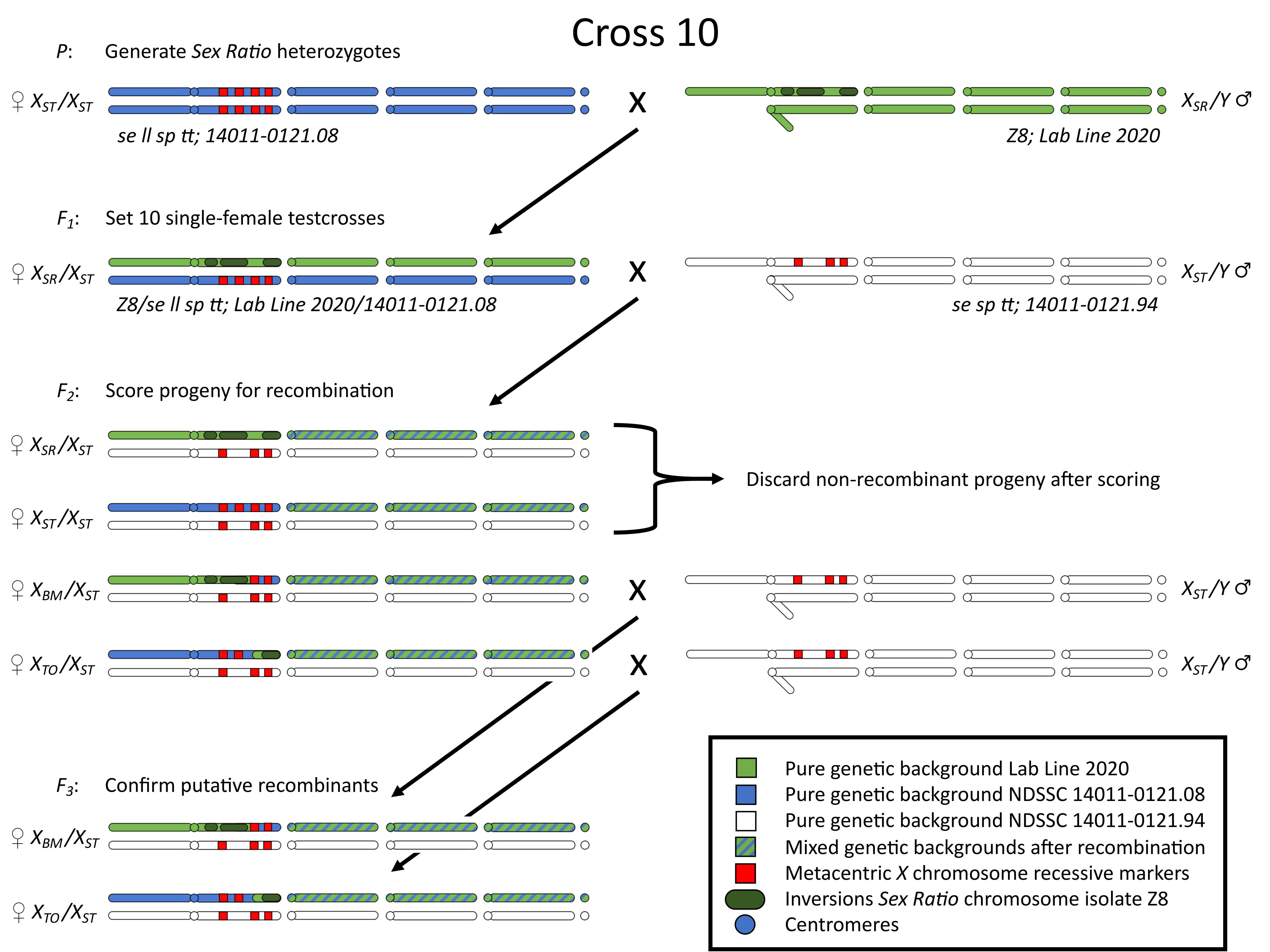

Cross 10
P: Generate Sex Ratio heterozygotes
♀ XST/XST
se ll sp tt; 14011-0121.08
X
XSR/Y ♂
Z8; Lab Line 2020
F1: Set 10 single-female testcrosses
♀ XSR/XST
Z8/se ll sp tt; Lab Line 2020/14011-0121.08
X
XST/Y ♂
se sp tt; 14011-0121.94
F2: Score progeny for recombination
♀ XSR/XST
♀ XST/XST
Discard non-recombinant progeny after scoring
♀ XBM/XST
X
XST/Y ♂
♀ XTO/XST
X
XST/Y ♂
F3: Confirm putative recombinants
♀ XBM/XST
♀ XTO/XST
Pure genetic background Lab Line 2020
Pure genetic background NDSSC 14011-0121.08
Pure genetic background NDSSC 14011-0121.94
Mixed genetic backgrounds after recombination
Metacentric X chromosome recessive markers
Inversions Sex Ratio chromosome isolate Z8
Centromeres

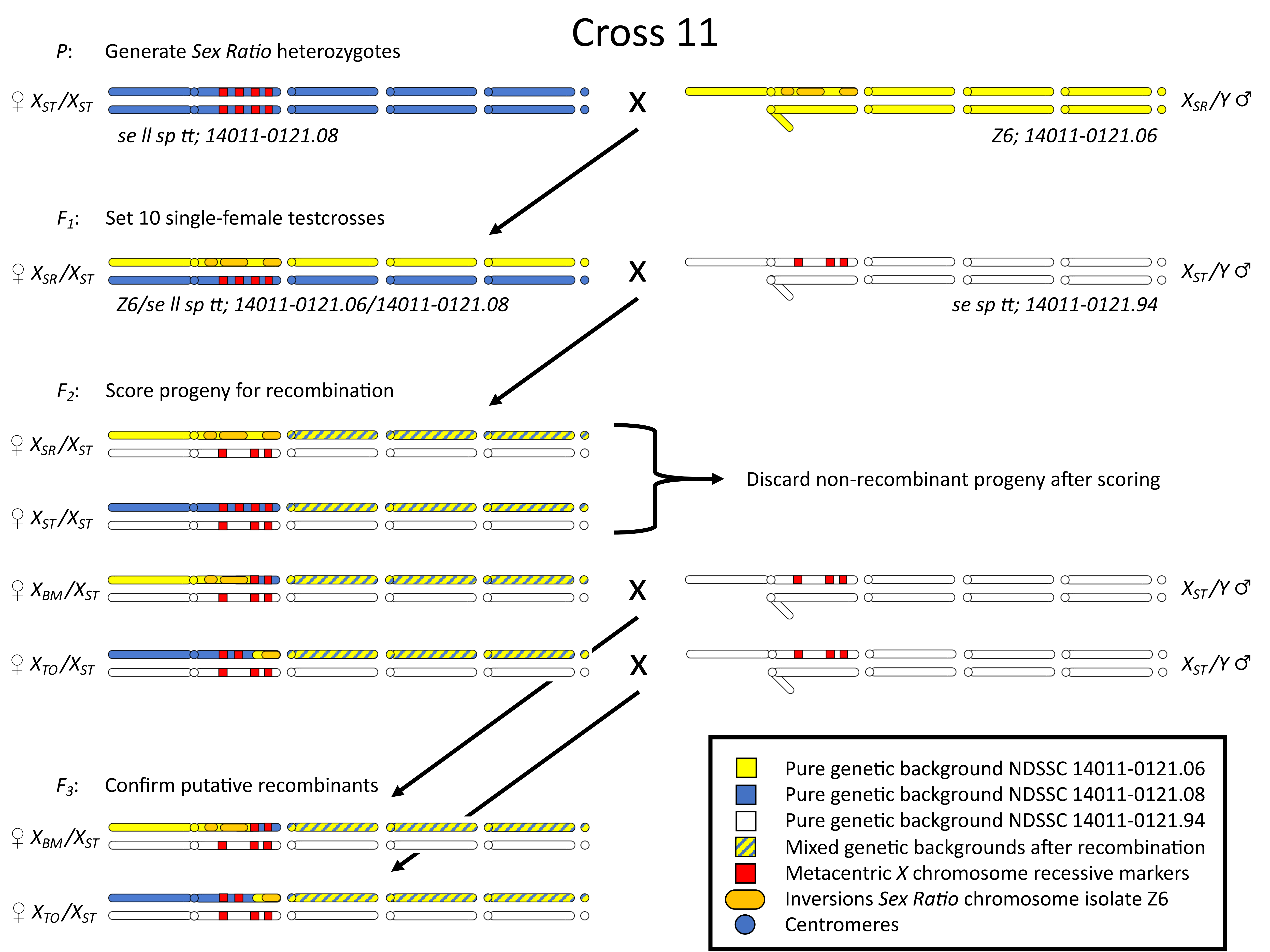
Cross 11
P: Generate *Sex Ratio* heterozygotes
♀ X_{ST}/X_{ST}
se ll sp tt; 14011-0121.08
X
X_{SR}/Y ♂
Z6; 14011-0121.06
F1: Set 10 single-female testcrosses
♀ X_{SR}/X_{ST}
Z6/se ll sp tt; 14011-0121.06/14011-0121.08
X
X_{ST}/Y ♂
se sp tt; 14011-0121.94
F2: Score progeny for recombination
♀ X_{SR}/X_{ST}
♀ X_{ST}/X_{ST}
Discard non-recombinant progeny after scoring
♀ X_{BM}/X_{ST}
X
X_{ST}/Y ♂
♀ X_{TO}/X_{ST}
X
X_{ST}/Y ♂
F3: Confirm putative recombinants
♀ X_{BM}/X_{ST}
♀ X_{TO}/X_{ST}
Pure genetic background NDSSC 14011-0121.06
Pure genetic background NDSSC 14011-0121.08
Pure genetic background NDSSC 14011-0121.94
Mixed genetic backgrounds after recombination
Metacentric *X* chromosome recessive markers
Inversions *Sex Ratio* chromosome isolate Z6
Centromeres

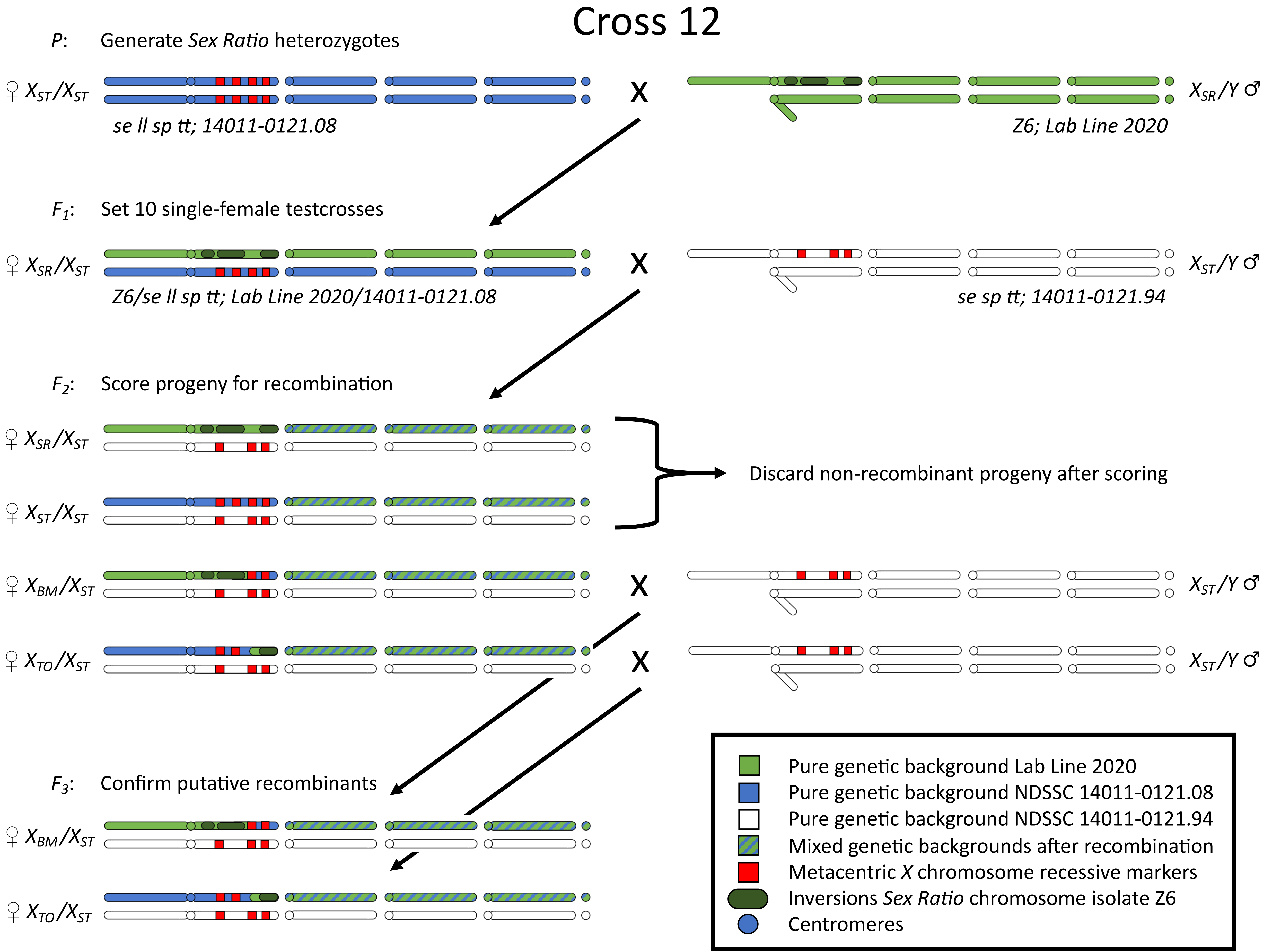
Cross 12
P: Generate Sex Ratio heterozygotes
♀ XST/XST
se ll sp tt; 14011-0121.08
X
XSR/Y ♂
Z6; Lab Line 2020
F1: Set 10 single-female testcrosses
♀ XSR/XST
Z6/se ll sp tt; Lab Line 2020/14011-0121.08
X
XST/Y ♂
se sp tt; 14011-0121.94
F2: Score progeny for recombination
♀ XSR/XST
♀ XST/XST
Discard non-recombinant progeny after scoring
♀ XBM/XST
X
XST/Y ♂
♀ XTO/XST
X
XST/Y ♂
F3: Confirm putative recombinants
♀ XBM/XST
♀ XTO/XST
Pure genetic background Lab Line 2020
Pure genetic background NDSSC 14011-0121.08
Pure genetic background NDSSC 14011-0121.94
Mixed genetic backgrounds after recombination
Metacentric X chromosome recessive markers
Inversions Sex Ratio chromosome isolate Z6
Centromeres

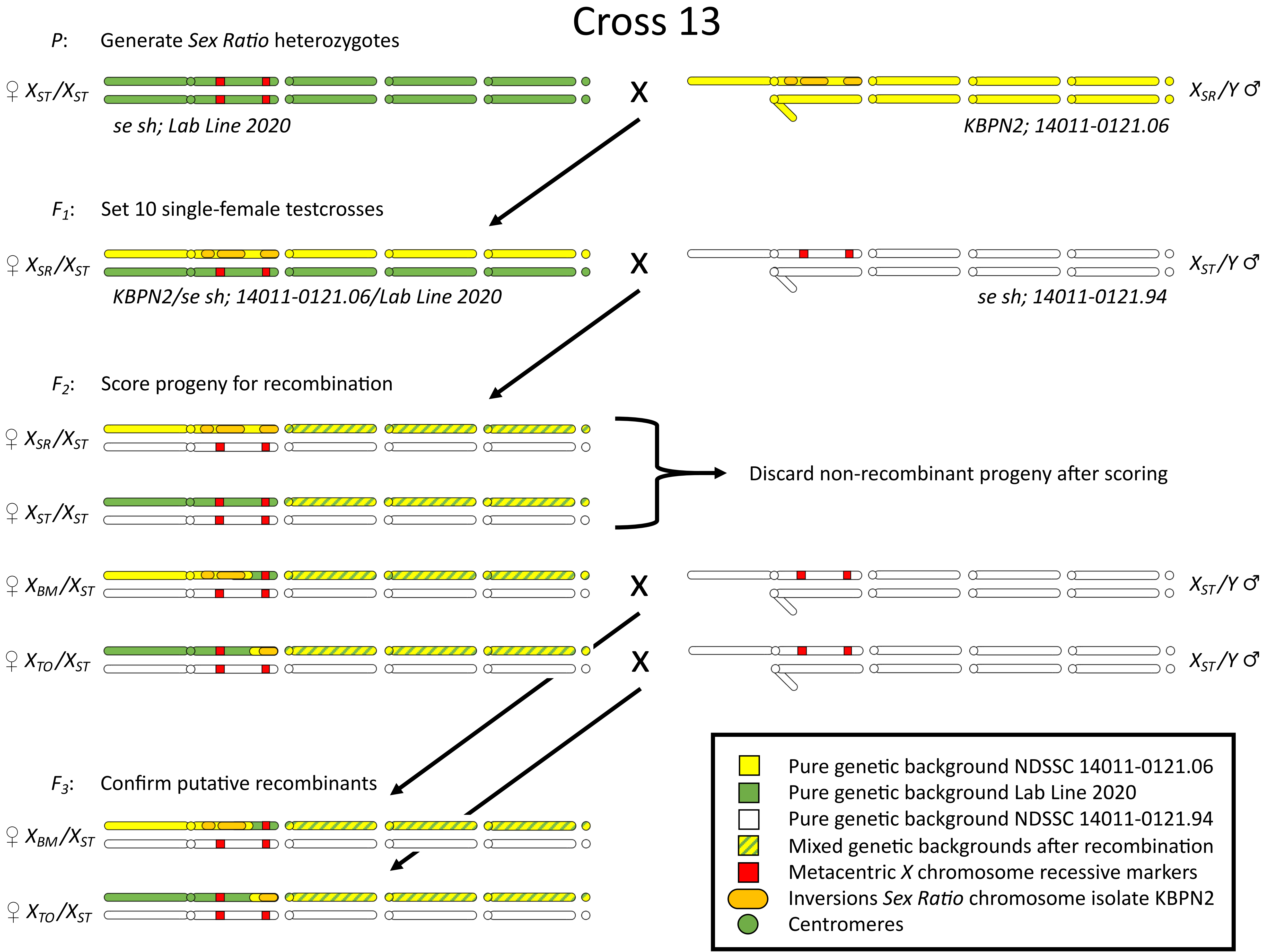

Cross 13
P: Generate Sex Ratio heterozygotes
♀ X_ST/X_ST
se sh; Lab Line 2020
X
X_SR/Y ♂
KBPN2; 14011-0121.06
F1: Set 10 single-female testcrosses
♀ X_SR/X_ST
KBPN2/se sh; 14011-0121.06/Lab Line 2020
X
X_ST/Y ♂
se sh; 14011-0121.94
F2: Score progeny for recombination
♀ X_SR/X_ST
♀ X_ST/X_ST
Discard non-recombinant progeny after scoring
♀ X_BM/X_ST
X
X_ST/Y ♂
♀ X_TO/X_ST
X
X_ST/Y ♂
F3: Confirm putative recombinants
♀ X_BM/X_ST
♀ X_TO/X_ST
Pure genetic background NDSSC 14011-0121.06
Pure genetic background Lab Line 2020
Pure genetic background NDSSC 14011-0121.94
Mixed genetic backgrounds after recombination
Metacentric X chromosome recessive markers
Inversions Sex Ratio chromosome isolate KBPN2
Centromeres

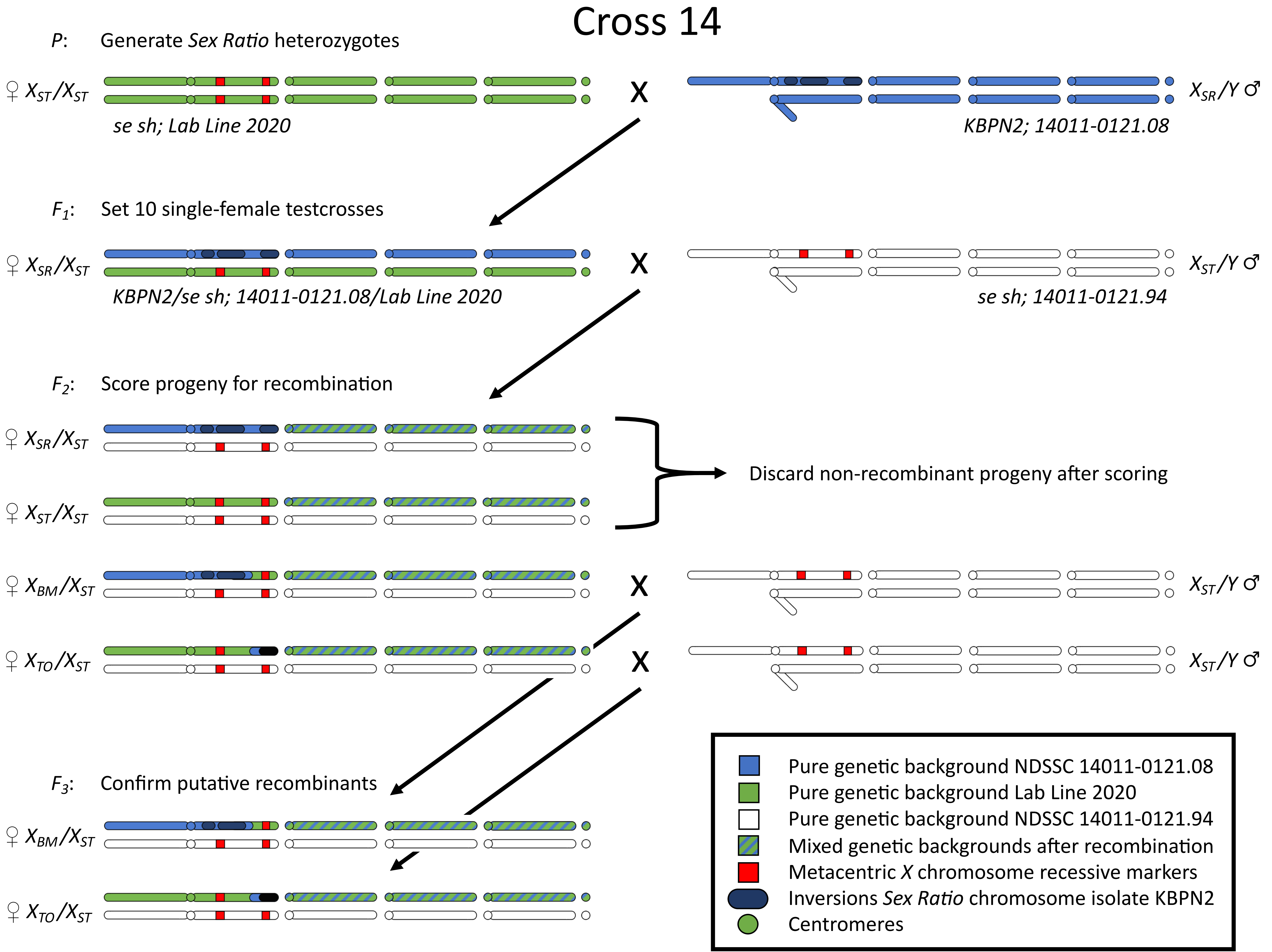
Cross 14
P: Generate Sex Ratio heterozygotes
♀ X_{ST}/X_{ST}
se sh; Lab Line 2020
X
X_{SR}/Y ♂
KBPN2; 14011-0121.08
F1: Set 10 single-female testcrosses
♀ X_{SR}/X_{ST}
KBPN2/se sh; 14011-0121.08/Lab Line 2020
X
X_{ST}/Y ♂
se sh; 14011-0121.94
F2: Score progeny for recombination
♀ X_{SR}/X_{ST}
♀ X_{ST}/X_{ST}
Discard non-recombinant progeny after scoring
♀ X_{BM}/X_{ST}
X
X_{ST}/Y ♂
♀ X_{TO}/X_{ST}
X
X_{ST}/Y ♂
F3: Confirm putative recombinants
♀ X_{BM}/X_{ST}
♀ X_{TO}/X_{ST}
Pure genetic background NDSSC 14011-0121.08
Pure genetic background Lab Line 2020
Pure genetic background NDSSC 14011-0121.94
Mixed genetic backgrounds after recombination
Metacentric X chromosome recessive markers
Inversions Sex Ratio chromosome isolate KBPN2
Centromeres

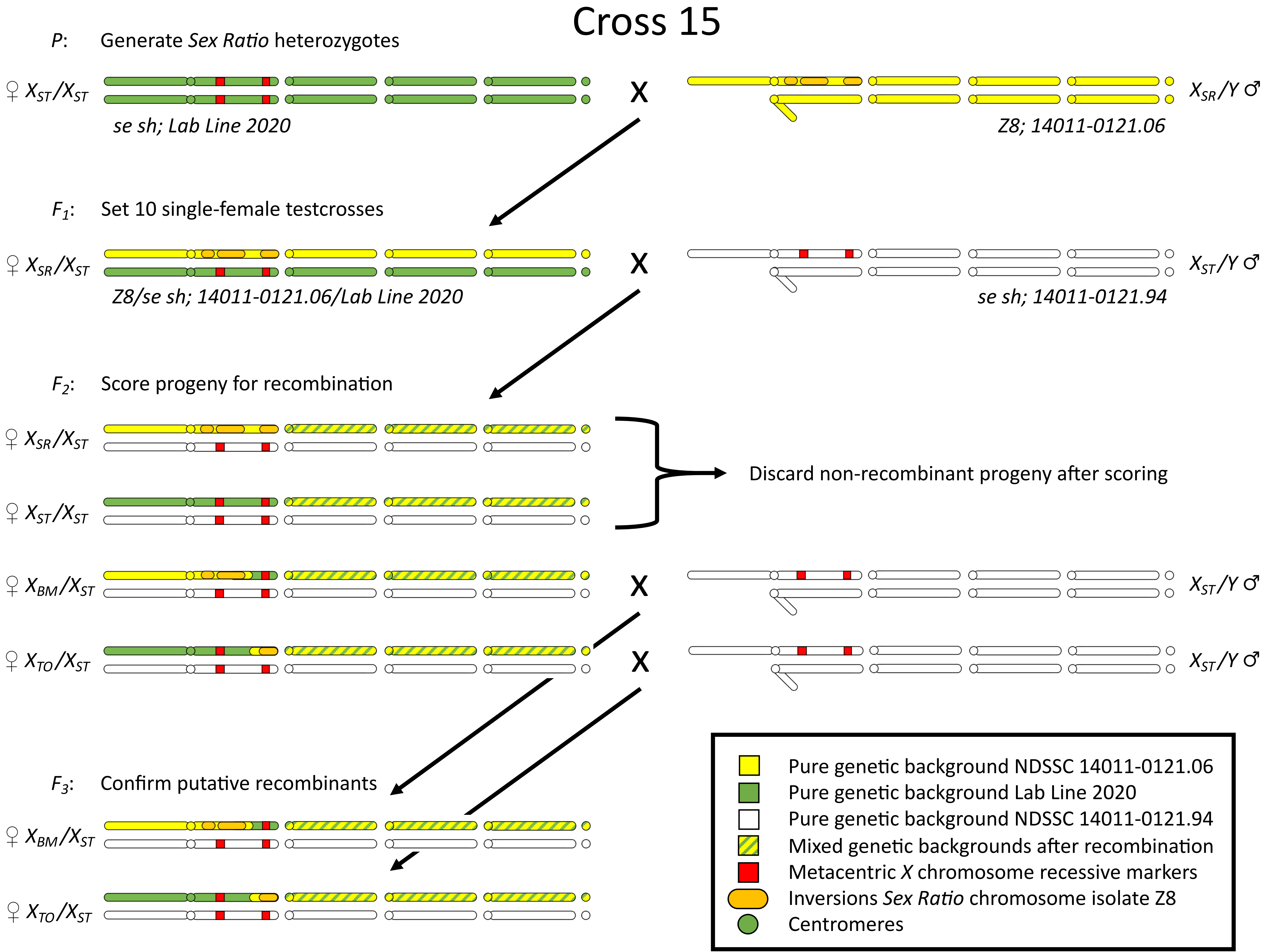

Cross 15
P: Generate Sex Ratio heterozygotes
♀ X_ST/X_ST
se sh; Lab Line 2020
X
Z8; 14011-0121.06
X_SR/Y ♂
F1: Set 10 single-female testcrosses
♀ X_SR/X_ST
Z8/se sh; 14011-0121.06/Lab Line 2020
X
se sh; 14011-0121.94
X_ST/Y ♂
F2: Score progeny for recombination
♀ X_SR/X_ST
♀ X_ST/X_ST
Discard non-recombinant progeny after scoring
♀ X_BM/X_ST
X
X_ST/Y ♂
♀ X_TO/X_ST
X
X_ST/Y ♂
F3: Confirm putative recombinants
♀ X_BM/X_ST
♀ X_TO/X_ST
Pure genetic background NDSSC 14011-0121.06
Pure genetic background Lab Line 2020
Pure genetic background NDSSC 14011-0121.94
Mixed genetic backgrounds after recombination
Metacentric X chromosome recessive markers
Inversions Sex Ratio chromosome isolate Z8
Centromeres

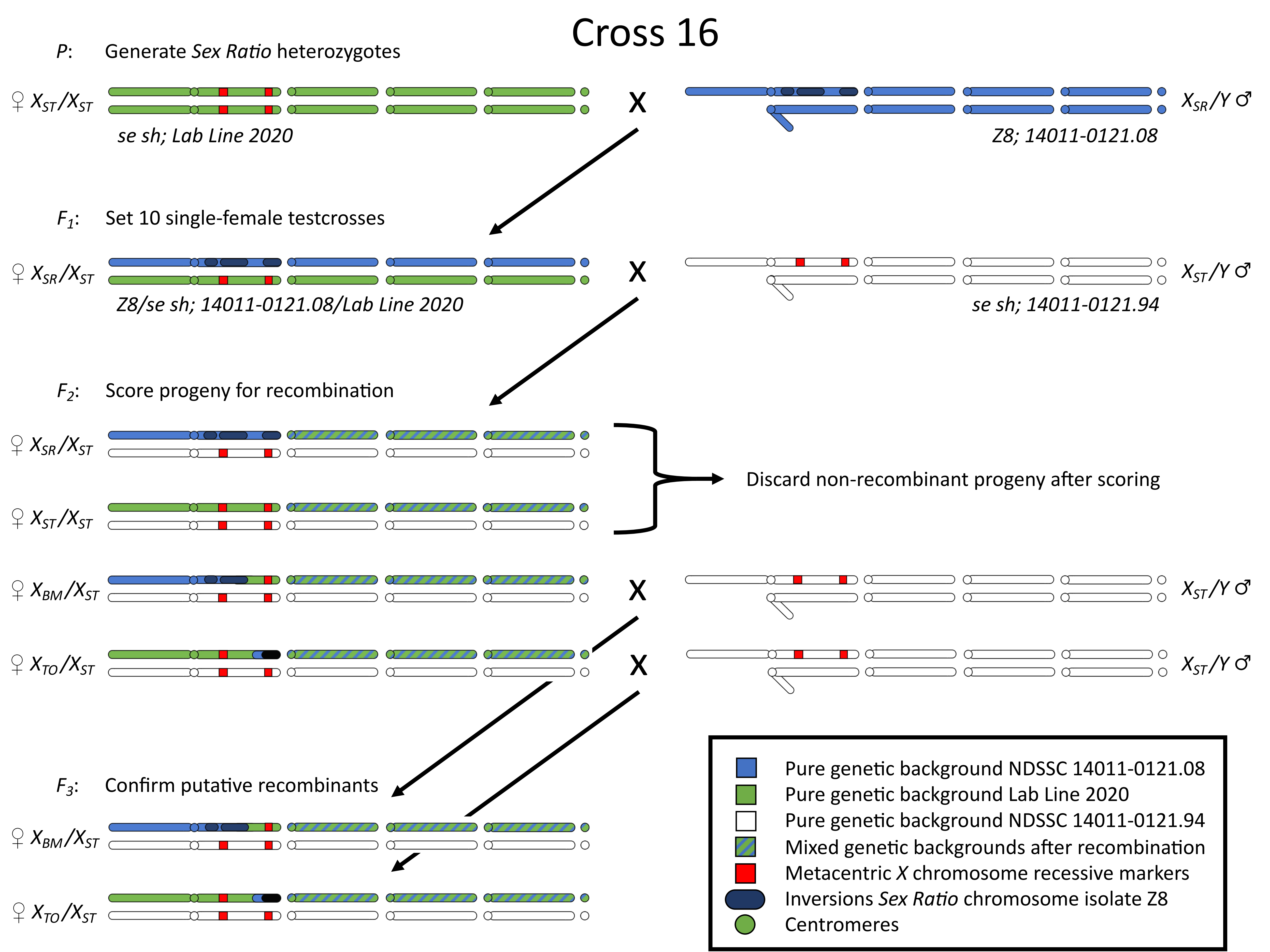

Cross 16
P: Generate Sex Ratio heterozygotes
♀ X_{ST}/X_{ST}
se sh; Lab Line 2020
X
X_{SR}/Y ♂
Z8; 14011-0121.08
F1: Set 10 single-female testcrosses
♀ X_{SR}/X_{ST}
Z8/se sh; 14011-0121.08/Lab Line 2020
X
X_{ST}/Y ♂
se sh; 14011-0121.94
F2: Score progeny for recombination
♀ X_{SR}/X_{ST}
♀ X_{ST}/X_{ST}
Discard non-recombinant progeny after scoring
♀ X_{BM}/X_{ST}
X
X_{ST}/Y ♂
♀ X_{TO}/X_{ST}
X
X_{ST}/Y ♂
F3: Confirm putative recombinants
♀ X_{BM}/X_{ST}
♀ X_{TO}/X_{ST}
Pure genetic background NDSSC 14011-0121.08
Pure genetic background Lab Line 2020
Pure genetic background NDSSC 14011-0121.94
Mixed genetic backgrounds after recombination
Metacentric X chromosome recessive markers
Inversions Sex Ratio chromosome isolate Z8
Centromeres

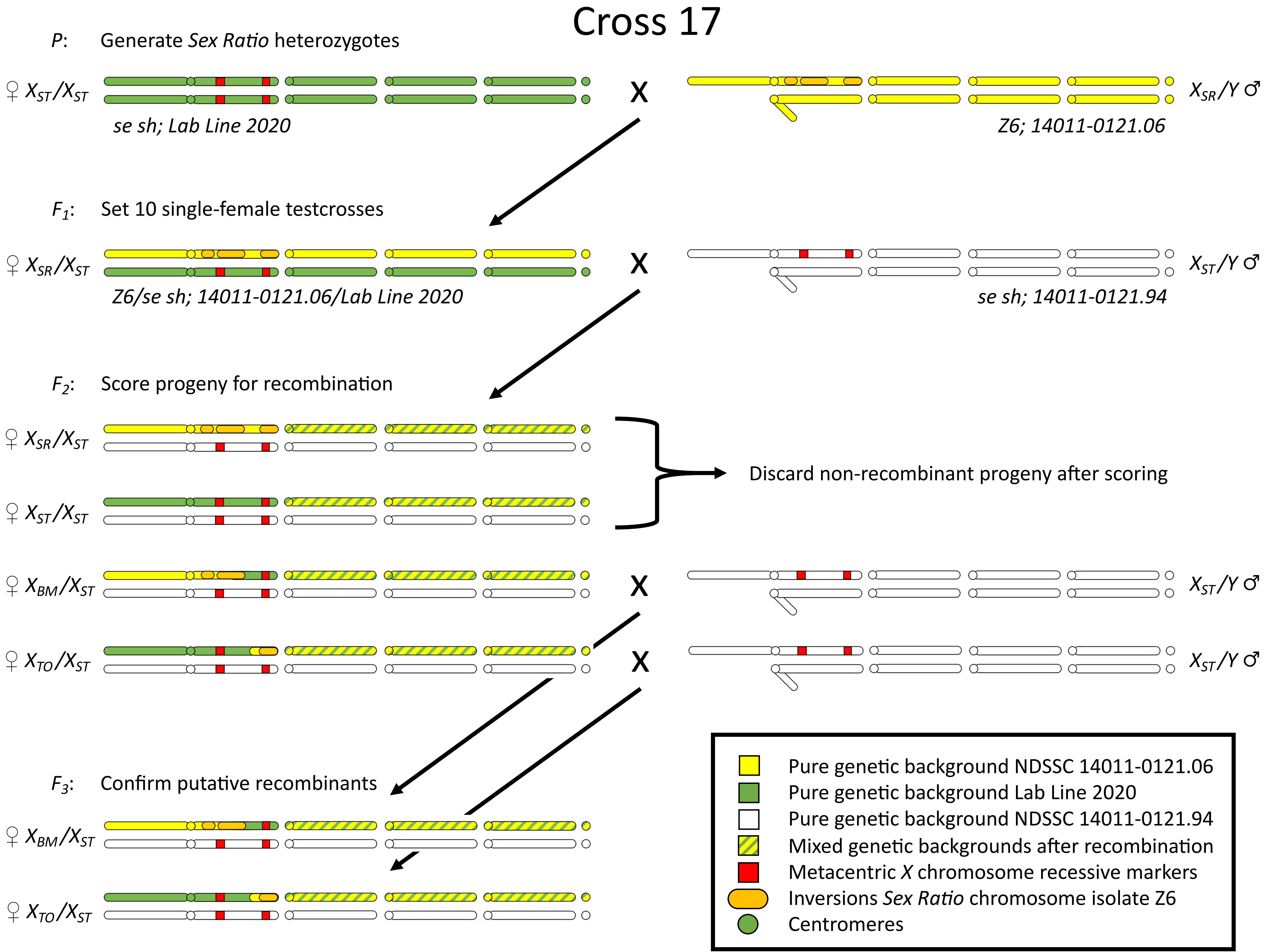
Cross 17
P: Generate Sex Ratio heterozygotes
♀ X_ST/X_ST
se sh; Lab Line 2020
X
X_SR/Y ♂
Z6; 14011-0121.06
F1: Set 10 single-female testcrosses
♀ X_SR/X_ST
Z6/se sh; 14011-0121.06/Lab Line 2020
X
X_ST/Y ♂
se sh; 14011-0121.94
F2: Score progeny for recombination
♀ X_SR/X_ST
♀ X_ST/X_ST
Discard non-recombinant progeny after scoring
♀ X_BM/X_ST
X
X_ST/Y ♂
♀ X_TO/X_ST
X
X_ST/Y ♂
F3: Confirm putative recombinants
♀ X_BM/X_ST
♀ X_TO/X_ST
Pure genetic background NDSSC 14011-0121.06
Pure genetic background Lab Line 2020
Pure genetic background NDSSC 14011-0121.94
Mixed genetic backgrounds after recombination
Metacentric X chromosome recessive markers
Inversions Sex Ratio chromosome isolate Z6
Centromeres

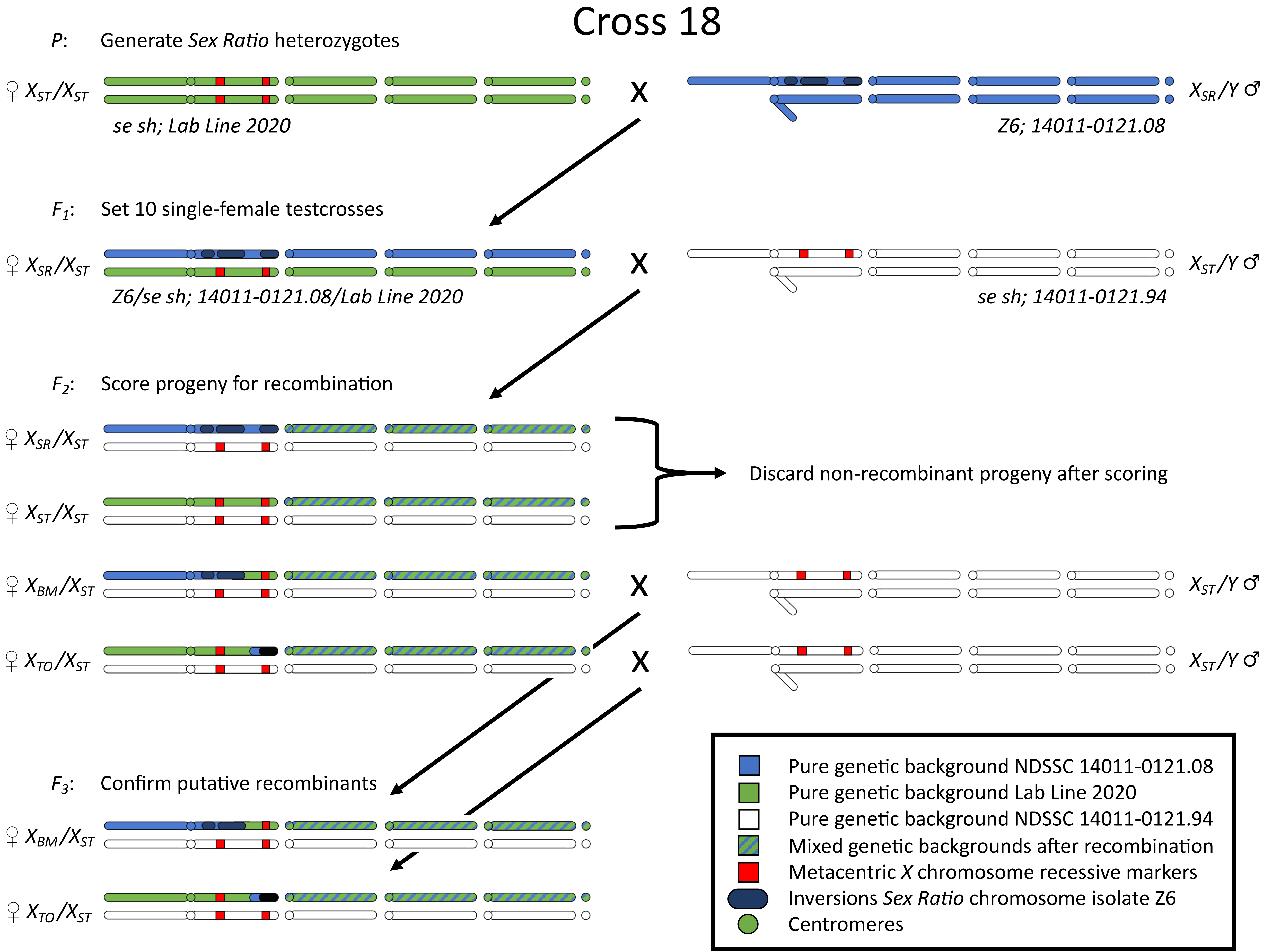
Cross 18
P: Generate Sex Ratio heterozygotes
♀ XST/XST
se sh; Lab Line 2020
X
XSR/Y ♂
Z6; 14011-0121.08
F1: Set 10 single-female testcrosses
♀ XSR/XST
Z6/se sh; 14011-0121.08/Lab Line 2020
X
XST/Y ♂
se sh; 14011-0121.94
F2: Score progeny for recombination
♀ XSR/XST
♀ XST/XST
Discard non-recombinant progeny after scoring
♀ XBM/XST
X
XST/Y ♂
♀ XTO/XST
X
XST/Y ♂
F3: Confirm putative recombinants
♀ XBM/XST
♀ XTO/XST
Pure genetic background NDSSC 14011-0121.08
Pure genetic background Lab Line 2020
Pure genetic background NDSSC 14011-0121.94
Mixed genetic backgrounds after recombination
Metacentric X chromosome recessive markers
Inversions Sex Ratio chromosome isolate Z6
Centromeres
